# Role of NMDA Receptors in Adult Neurogenesis and Normal Development of the Dentate Gyrus

**DOI:** 10.1523/ENEURO.0566-20.2021

**Published:** 2021-08-04

**Authors:** Ingrid Åmellem, Glen Yovianto, Hai Tarng Chong, Rajeevkumar Raveendran Nair, Vanja Cnops, Ayesha Thanawalla, Ayumu Tashiro (田代 歩)

**Affiliations:** 1 School of Biological Sciences, Nanyang Technological University, Singapore 308232; 2 Kavli Institute for Systems Neuroscience, Norwegian University of Science and Technology, Trondheim 7491, Norway

**Keywords:** granule cell, hippocampus, neuronal survival, novelty suppressed feeding test, pattern separation

## Abstract

The NMDA receptors are a type of glutamate receptors, which is involved in neuronal function, plasticity and development in the mammalian brain. However, how the NMDA receptors contribute to adult neurogenesis and development of the dentate gyrus is unclear. In this study, we investigate this question by examining a region-specific knock-out mouse line that lacks the NR1 gene, which encodes the essential subunit of the NMDA receptors, in granule cells of the dentate gyrus (DG-NR1KO mice). We found that the survival of newly-generated granule cells, cell proliferation and the size of the granule cell layer are significantly reduced in the dorsal dentate gyrus of adult DG-NR1KO mice. Our results also show a significant reduction in the number of immature neurons and in the volume of the granule cell layer, starting from three weeks of postnatal age. DG-NR1KO mice also showed impairment in the expression of an immediate early gene, Arc, and behavior during the novelty-suppressed feeding and open field test. These results suggest that the NMDA receptors in granule cells have a role in adult neurogenesis in the adult brain and contributes to the normal development of the dentate gyrus.

## Significance Statement

A type of glutamate receptors, the NMDA receptors, are known to be generally important in brain development. However, a previous study, which genetically ablated the NMDA receptors in the developing dentate gyrus, did not detect malformation of the dentate gyrus. Here, we performed more detailed analyses of the knock-out mice and showed that the extent of neurogenesis and the size of the neuronal layer are reduced in the postnatal and adult dentate gyrus. We found that the knock-out mice exhibit behavioral abnormality during adulthood. These results indicate that the NMDA receptor is essential for normal development of the dentate gyrus and that a malfunction of the NMDA receptors during the postnatal period could lead to life-long disturbance in brain functions.

## Introduction

A type of ionotropic glutamate receptors, the NMDA receptors, are involved in postnatal brain development (Haberny et al., 2002; Hansen et al., 2004; Ewald and Cline, 2009). It has been shown that the NMDA receptors support the survival of developing neurons (Ikonomidou et al., 1999) and regulate the formation of neuronal networks in several brain areas (Bear et al., 1987; Constantine-Paton et al., 1990), including the dentate gyrus (Gould et al., 1994). The majority of granule cells, a principal cell type in the dentate gyrus, are generated postnatally, predominantly during the first few weeks after birth in rodents (Cameron et al., 1993) and the structural development of the dentate gyrus occurs mainly during the first month of the postnatal period in rodents (Li and Pleasure, 2005). Early life experience, such as stress, has been found to regulate the expression of the NMDA receptors in the hippocampus (Roceri et al., 2002; Pickering et al., 2006) and to reduce the number of granule cells (Oreland et al., 2010).

The involvement of the NMDA receptors in neuronal survival and circuit formation continues in newly-generated neurons in the adult brain. The survival of newborn neurons in the adult brain is regulated by neuronal activity in the dentate gyrus, as evidenced by the finding that induction of NMDA receptor-dependent long-term potentiation facilitates the survival of newborn neurons (Bruel-Jungerman et al., 2006). A study using a cell-specific gene knock-out technique (Tashiro et al., 2006a) demonstrated that the NMDA receptors positively regulate the survival of immature granule cells in a cell-autonomous manner, within a few weeks of neuronal birth (Tashiro et al., 2006b). However, once new granule cells pass a certain maturational stage, the NMDA receptors are not required for their survival but continue to be required for the regulation of dendritic spine morphology and synaptic plasticity (Ge et al., 2007; Mu et al., 2015).

A previous study demonstrated that the NMDA receptors in granule cells of the dentate gyrus play a role in a context discrimination task using a region-specific gene knock-out mouse line. This mouse line lacks the NR1 gene, which encodes an essential subunit of the NMDA receptors, specifically in granule cells of the dentate gyrus (McHugh et al., 2007). The gene knock-out in granule cells starts between 1.5 and 4 weeks after the birth of mice. The study found that the gross structure of the dentate gyrus is intact but did not examine how adult neurogenesis is affected in the mouse line.

In this study, we have two aims. The first aim is to determine a role of NMDA receptors in adult neurogenesis of the dentate gyrus. We used the same knock-out mouse line created by McHugh et al. (2007; DG-NR1KO mice) and examined neurogenesis in the adult dentate gyrus. The second aim is to reveal the role of NMDA receptors in normal formation of the dentate gyrus. As described above, the NMDA receptors have been shown to contribute to the development of granule cells in the postnatal and adult dentate gyrus. However, McHugh et al. (2007) did not detect that the NR1 gene knock-out starting from the postnatal development affects the development of the dentate gyrus. To reconcile this discrepancy in the contribution of the NMDA receptors to the development of the dentate gyrus, we examined the structure and neurogenesis in the dentate gyrus of the postnatal and adult brain.

## Materials and Methods

### Mice

**Figure 1.**
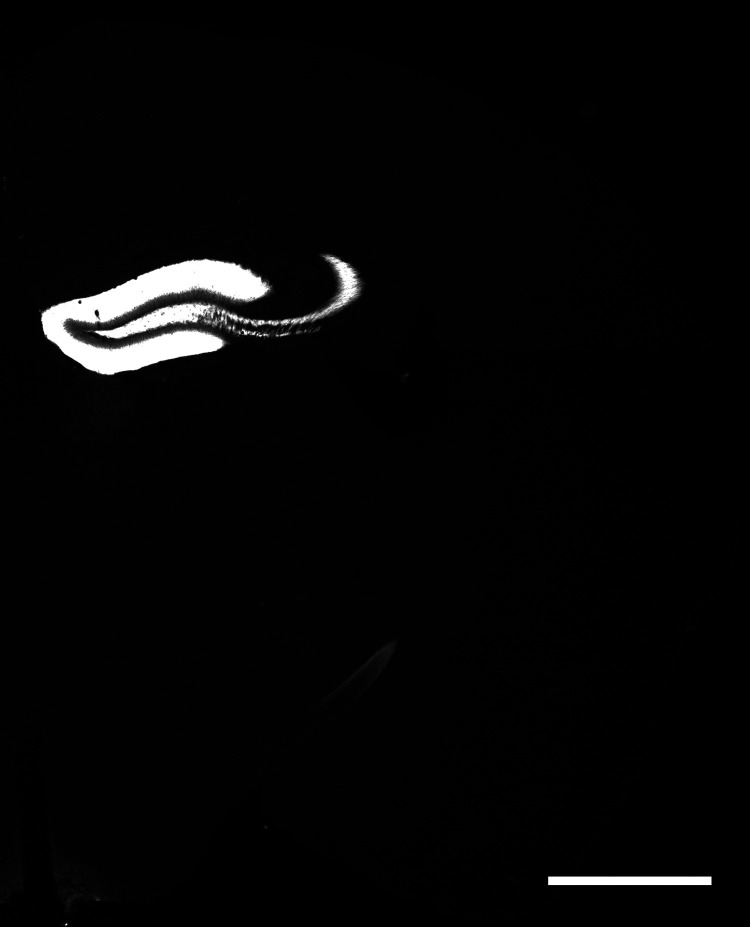
Specificity of Cre expression. A representative image showing YFP signal in Cre+/− offspring from crossing Pomc-cre+/− and cre-dependent ChR2-YFP reporter lines. Consistent with McHugh et al. (2007), we found dense expression in the dentate gyrus and sparser expression in the arcuate nucleus of the hypothalamus and the habenula. We also noted sparse expression in the optic tract, which McHugh et al. (2007) did not mention, but we can observe it in their figures (McHugh et al., 2007; their Fig. 1*A*,*D*). Fluorescence signal in the molecular layer of the dentate gyrus and stratum lucidum of the CA3 area reflects the dendrites and axons of granule cells, respectively. Scale bar: 1 mm.

All animal procedures were approved by the Norwegian Animal Research Authority and/or Institutional animal care and use committee at A*STAR and Nanyang Technological University. We re-generated DG-NR1KO (Pomc-cre+/−, fNR1+/+) mice using the same transgenic mouse lines used by McHugh et al. (2007), B6.FVB-Tg(Pomc-cre)1Stl/J and B6.129S4-Grin1P^tm2Stl^P/J (fNR1; both from The Jackson Laboratory). Cre recombinase is under transcriptional control by the proopiomelanocortin (Pomc) promoter, which initiates gene expression during a short time window in the maturation of newborn granule cells of the dentate gyrus (Overstreet et al., 2004). Specificity of Cre expression in Pomc-cre+/− mice was equivalent with McHugh et al. (2007) when we evaluated it by crossing them with a cre-dependent reporter line (B6;129S-Gt(ROSA)26Sor^tm32^^(CAG-COP4*H134R/EYFP)Hze^/J, from The Jackson Laboratory; Fig. 1). Cre recombines a floxed part of the NR1 gene (fNR1) to ablate the functional NR1 gene. These mice were bred with Pomc-cre−/−, fNR1+/+ mice, and their Cre+/− offspring were used as DG-NR1KO mice and the Cre-negative littermates were used as controls. Both female and male mice were used except Figure 6, where we used female mice only. For Figures 3, 4, 8*G–O*, we used young adult mice (42–51 d old at the start of experiments). For Figure 5, we used two-, three-, or four-week-old mice. For Figures 6, 7, 8*A–F*, we used young adult to mature adult mice (42–83 d old). Mice were housed with *ad libitum* access to food and water under 12/12 h light/dark cycle conditions, unless otherwise specified below.

### Genotyping

The genotypes of mice were determined by PCR analyses. The primer sequences for Cre are 5′-CCGGTGAACGTGCAAAACAGGCTCTA-3′ and 5′-GATTAACATTCTCCCACCGTCAGT-3′ and those for the fNR1 allele are 5′-CTTGGGTGGAGAGGCTATTC-3′ and 5′-AGGTGAGATGACAGGAGATC-3′. As previously described (McHugh et al., 2007), germline recombination occurs in this mouse line. To detect recombined alleles in control mice, nested PCR was performed. The primer sequences for the initial amplification were 5′-AATGCTGAGGTGGTAGGA-3′ and 5′-AGGTGAGATGACAGGAGATC-3′. The primer sequences for the second step are 5′-GCTACAAGGCAAAGATACAAGACC-3′ and 5′-ACCGTCGACGAGAATTCCGATCAT-3′. When a recombined allele was detected in control mice, they were excluded from the experiments.

### 5-Bromo-2’-deoxyuridine (BrdU) injections

BrdU (catalog #B5002, Sigma) was dissolved in 0.9% saline at a concentration of 10 mg/ml. After filter sterilization (pore size: 0.2 μm), mice were intraperitoneally injected with a dose of 100 μg/g body weight per day.

### Enriched environment

**Figure 2.**
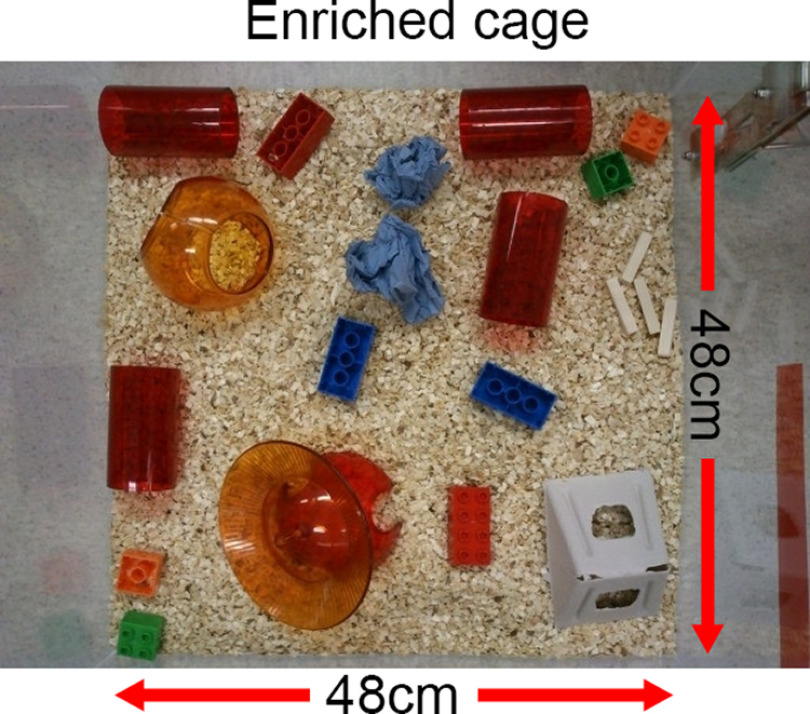
Enriched housing used in this study.

Female mice were injected with BrdU on days −1 and 0. Between days 7 and 14, they were either housed in an enriched cage (48 × 48 × 48 cm, eight mice per cage) which contained eight pieces of plastic blocks, one cardboard shelter, one plastic shelter, four tunnels, two sheets of paper for nesting, four pieces of small wood, and one running wheel (Fig. 2), or they stayed in the standard housing (26.7 × 20.7 × 14 cm, four mice per cage) with bedding only. On days 0–7 and days 14–28, all the mice were in the standard housing. The mice were perfusion-fixed on day 28 as described below.

### Novel environment exploration

The mice were exposed to a novel environment, which was a 32 × 28 × 28 cm open field with plastic walls with black and white stripes. They were allowed to explore the novel environment for 10 min. Then, the mice were moved back to their home cages in the experimental room. After 90 min, they were perfusion-fixated as described below. The non-exposed mice were taken directly from their home cage in the housing room for perfusion.

### Novelty suppressed feeding test

The mice were food deprived for 24 h before the test. A food pellet was placed on a white paper platform (12.5 cm) at the center of a square open field (60 × 60 cm, with white colored walls), which the mice had never been exposed to before. The room was lit with regular room lighting (around 700 lux at the center of the box) and the floor of the open field was covered with the same type of bedding as used in their home cages. At the start of a test, a mouse was placed at a corner of the open field. When the mouse started eating the food pellet or 10 min passed after the test started, the test was terminated. The test was recorded by an overhead camera and analyzed using EthoVision XT13 software (Noldus Information Technology BV). The area in the open field was divided into 5 × 5 square bins. A total of 16 outer bins were defined as the peripheral zone. The area on the white paper was defined as the center zone. The remaining area was defined as the inner zone.

### Open field test

A square open field (60 × 60 cm, with white-colored walls and floor) was placed in a room that was lit with ceiling lights (∼700 lux at the center of the box). At the start of a test, a mouse was placed at a corner of the open field and allowed to explore it. After 10 min, the test was terminated. The mice were removed from the open field and fecal boli were counted. The test was recorded by an overhead camera and analyzed using EthoVision XT13 software. The area in the open field was divided into 5 × 5 square bins. A total of 16 outer bins were defined as the peripheral zone. The remaining area was defined as the inner zone.

### Elevated plus maze

A plus shaped maze was elevated 60 cm above the ground. Two arms opposite to each other were surrounded with 15-cm-high walls at the side and end of the arm. These are called “closed arm” while the other two arms without walls were called “open arm.” The lengths of closed and open arms were 35 and 36.5 cm, respectively. The width of each arm was 5 cm. The room was lit dimly (∼100 lux at the center of the maze). At the start of a test, a mouse was placed at the center of the maze, with its face facing one of the closed arms. The mouse was allowed to explore the maze. After 10 min, the test was terminated. The test was recorded by an overhead camera and analyzed using Anymaze 6.34 (Stoelting Co). The entry to and exit from an arm were registered when 40% of body entered and 35% exited the arm, respectively.

### Histology

Perfusion fixation, preparation of brain sections (35- or 40-μm thickness) and immunostaining were conducted as previously described (Tashiro et al., 2015). We prepared coronal brain sections containing the rostral two thirds of the dentate gyrus; therefore, the analysis presented in this study is mostly from the dorsal, but not ventral, part of the dentate gyrus. The primary antibodies used were goat anti-DCX (1:500 dilution, catalog #sc-8066, Santa Cruz Biotechnology) and mouse anti-activity-regulated cytoskeleton-associated protein (Arc; 1:100, catalog #sc-17839, Santa Cruz Biotechnology), rat anti-GFP (1:1000, catalog #04404-84, Nacalai Tesque), rat anti-BrdU (1:400, catalog #OBT0030G, AbD Serotec), rabbit anti-Prospero homeobox protein 1 (Prox1; 1:1000, catalog #PRB-238C, Covance), goat anti-Prox1 (1:40, catalog #AF 2727, R&D Systems), and rabbit anti-Ki67 (1:500, catalog #NCL-Ki67p, Leica Biosystems). The secondary antibodies used were donkey anti-rat-Alexa Fluor 488 (catalog #712-545-153), anti-rabbit-Cy3 (catalog #712-165-152), anti-mouse-DyLight549 (catalog #715-505-151), anti-goat-Alexa Fluor 488 (catalog #705-545-147), and anti-goat-Alexa Fluor 647 (catalog #705-605-147). All secondary antibodies were purchased from Jackson ImmunoResearch and used at 1:600 dilutions. Nuclear staining with 4’,6-diamidino-2-phenylindole-dihydrochloride (DAPI; 5 μg/ml; Merck) was done on some of the sections. The brain sections were mounted on glass slides with mounting medium containing PVA-DABCO.

### Microscopy

**Figure 3.**
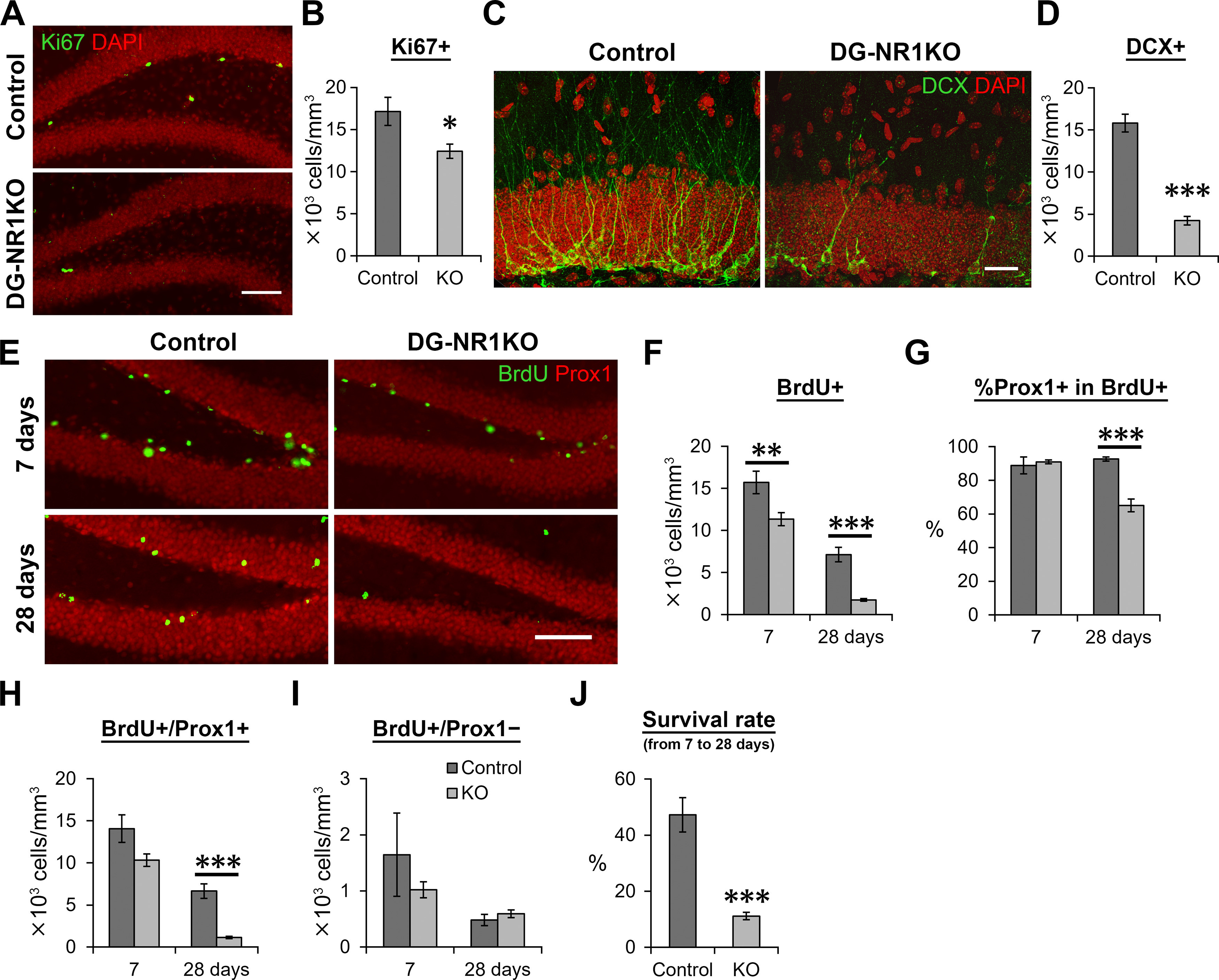
Cell proliferation and the survival of new neurons were reduced in the dentate gyrus of adult DG-NR1KO mice. ***A***, Representative images visualizing Ki67+ (green) and DAPI-labeled (red) cells in the dentate gyrus of adult control and DG-NR1KO mice. Scale bar: 75 μm. ***B***, Density of Ki67+ cells in the subgranular zone. ***C***, Representative images showing DCX+ (green) and DAPI-labeled (red) cells in the dentate gyrus of adult control and DG-NR1KO mice. The images were maximum intensity projections of confocal Z stacks and formed by joining two overlapping images containing adjacent areas. Scale bar: 25 μm. ***D***, Density of DCX+ cells. ***E***, Representative images showing BrdU+ (green) and Prox1+ (red) cells in adult control and DG-NR1KO mice on 7 and 28 d after BrdU injections. Scale bar: 75 μm. ***F***, Density of BrdU+ cells in the granule cell layer and subgranular zone. ***G***, Proportion of BrdU+ cells expressing Prox1. ***H***, Density of BrdU+/Prox1+ cells in the granule cell layer. ***I***, Density of BrdU+/Prox1– cells in the granule cell layer. ***J***, Survival rate of BrdU+/Prox1+ cells from 7 to 28 dpi. Density at 28 d after BrdU injection was divided by mean values of density at 7 d; *p < 0.05, **p < 0.01, ***p < 0.005, independent-sample *t* test, two tailed.

**Figure 4.**
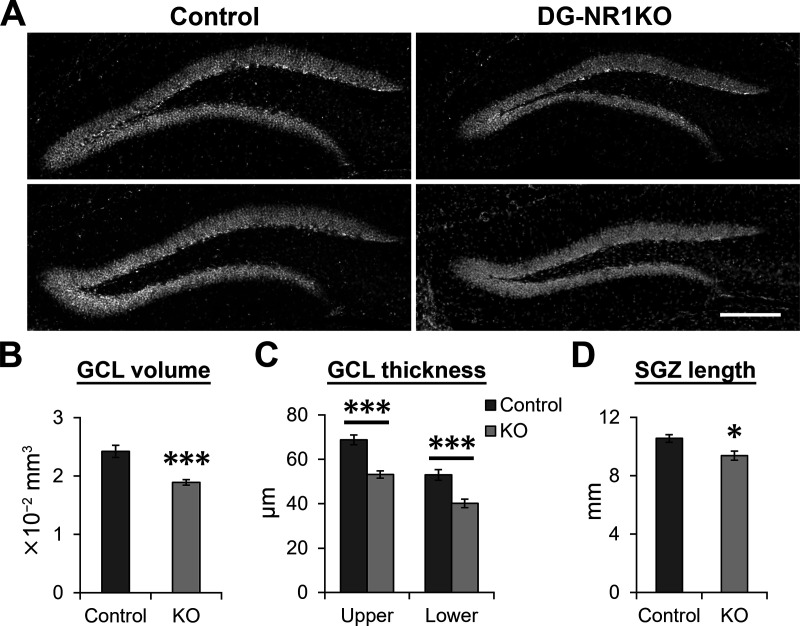
Reduced size of the granule cell layer in adult DG-NR1KO mice. ***A***, Representative images showing the granule cell layer visualized by DAPI staining in adult control and DG-NR1KO mice. Scale bar: 150 μm. ***B***, Volume of the granule cell layer (GCL) in adult control and DG-NR1KO mice. Summed volume from three analyzed sections is presented. ***C***, Thickness of the upper and lower blade of granule cell layer in adult control and DG-NR1KO mice. ***D***, Length of the subgranular zone (SGZ) in adult control and DG-NR1KO mice; *p < 0.05, ***p < 0.005, independent-sample *t* test, two tailed.

**Figure 5.**
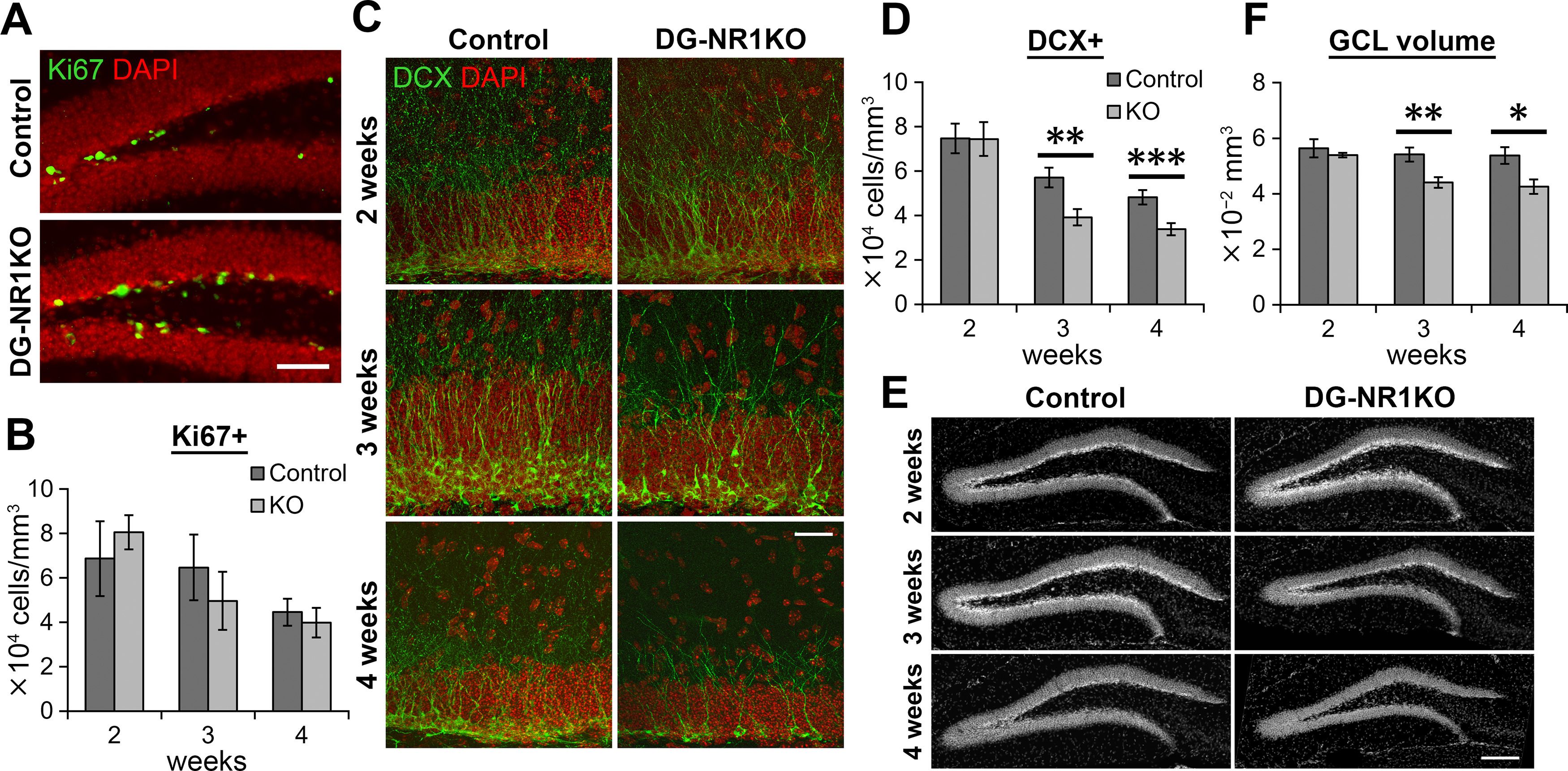
Neurogenesis was impaired in the dentate gyrus of postnatal, developing DG-NR1KO mice. ***A***, Representative images showing Ki67+ (green) and DAPI-labeled (red) cells in the dentate gyrus of four-week-old control and DG-NR1KO mice. Scale bar: 60 μm. ***B***, Density of Ki67+ cells in the subgranular zone in two-, three-, and four-week-old control and DG-NR1KO mice. ***C***, Representative images showing DCX+ (green) and DAPI-labeled (red) cells in the dentate gyrus of two-, three-, and four-week-old control and DG-NR1KO mice. The images were maximum intensity projections of confocal Z stacks. Scale bar: 25 μm. ***D***, Density of DCX+ cells in the granule cell layer of two-, three-, and four-week-old control and DG-NR1KO mice. ***E***, Representative images showing the granule cell layer visualized by DAPI staining in two-, three-, and four-week-old control and DG-NR1KO mice. Scale bar: 150 μm. ***F***, Volume of the granule cell layer (GCL) in two-, three-, and four-week-old control and DG-NR1KO mice. Summed volume from six analyzed sections is presented; *p < 0.05, **p < 0.01, ***p < 0.005, independent-sample *t* test, two tailed.

**Figure 6.**
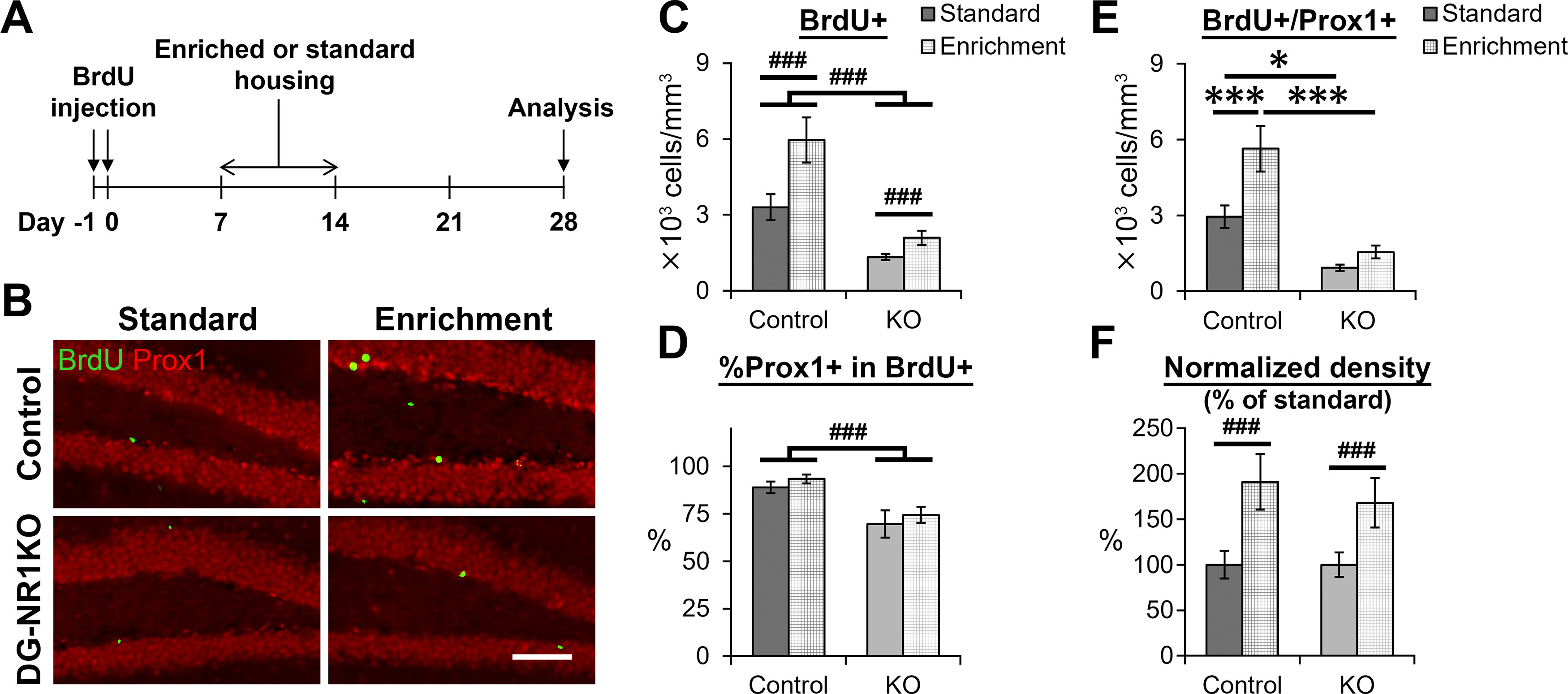
Exposure to an enriched environment increases the survival of newborn neurons in DG-NR1KO mice. ***A***, Experimental timeline. ***B***, Representative images showing BrdU+ (green) and Prox1+ (red) cells in the dentate gyrus of adult control and DG-NR1KO mice with or without exposure to the enriched environment. Scale bar: 60 μm. ***C***, Density of BrdU+ cells in the dentate gyrus of adult control and DG-NR1KO mice with or without exposure to the enriched environment. ***D***, Proportion of BrdU+ cells expressing Prox1. ***E***, Density of BrdU+/Prox1+ cells in the granule cell layer of adult control and DG-NR1KO mice with or without exposure to the enriched environment. ***F***, Normalized density of BrdU+/Prox1+ cells in the granule cell layer of adult control and DG-NR1KO mice with or without exposure to the enriched environment. Density of each mouse was divided by a mean value of mice without exposure to the enriched environment in the same genotype; *p < 0.05, ***p < 0.005, Tukey’s HSD test (performed because of significant genotype × housing interaction in two-way ANOVA); ###*p* < 0.005, the main effect of genotype or housing (without significant interaction).

Axio Scope A1 or Axio Imager M1 microscopes (Zeiss, Germany) were used to count BrdU-positive (+), Ki67+, and DCX+ cells and image the granule cell layer of the dentate gyrus for size measurements. Counting was done using either 20× or 40× objective lens from the granule cell layer and subgranular zone in both hemispheres in six brain sections per mouse for BrdU (every 6th section; Figs. 3, 6), three brain sections for Ki67 and DCX (every 12th section; Fig. 3) in adult mice, six brain sections from the anterior part of the dentate gyrus for Ki67 and granule cell layer volume measurements in two- to four-week-old mice (every 6th section; Fig. 5), and two brain sections from similar anterior part for DCX in two- to four-week-old mice (Fig. 5). The DAPI or Prox1 fluorescence were used to image the area of the granule cell layer using a 10× objective lens. The volume of the granule cell layer was calculated by multiplying the area by the thickness of the sections, which was 35 or 40 μm. The volume values shown in Figures 4, 5 are summed volume from three and six analyzed sections, respectively.

**Figure 7.**
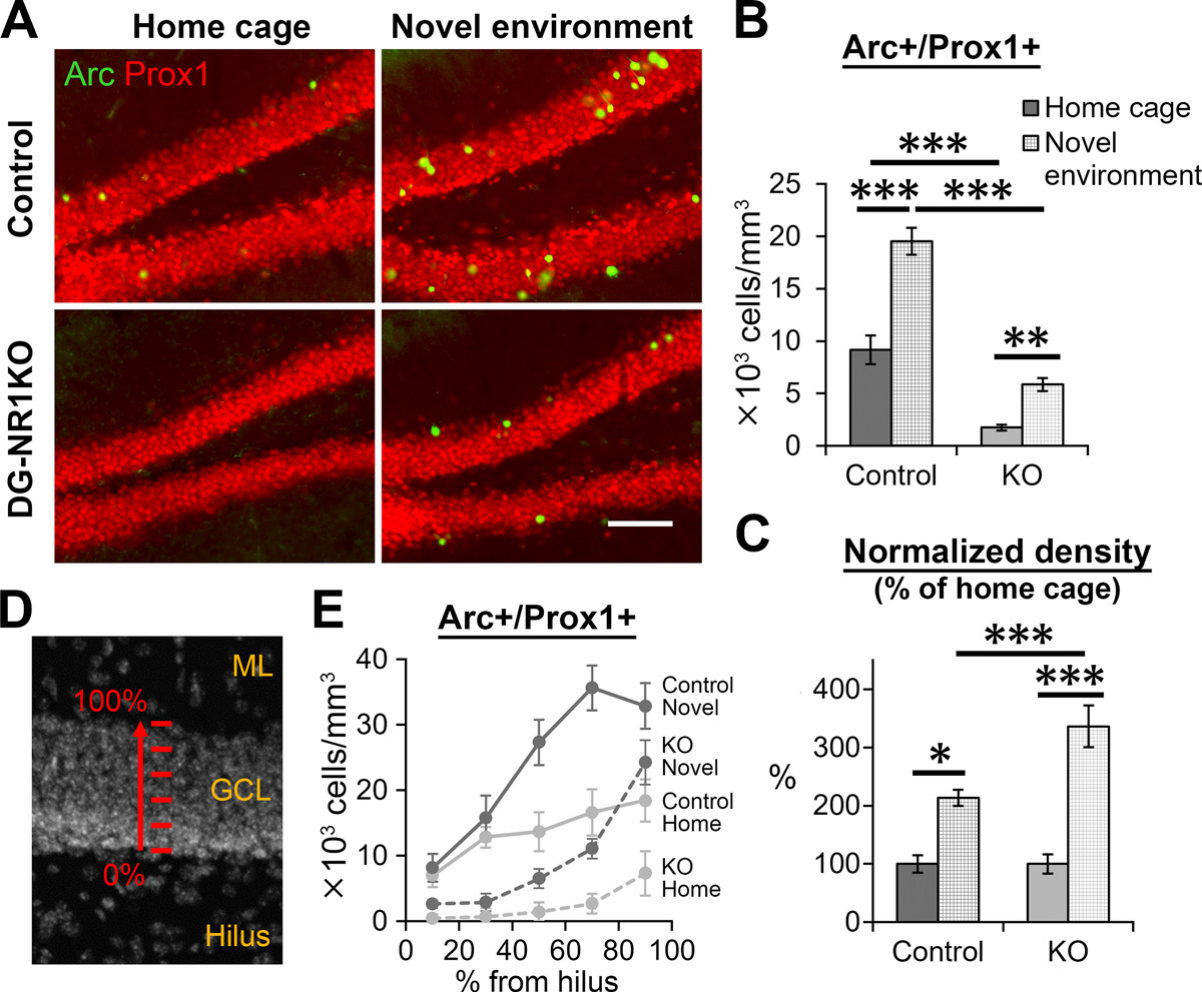
Impairment in novelty-induced Arc gene expression in granule cells of DG-NR1KO mice. ***A***, Representative images showing Arc+ (green) and Prox1+ (red) expressing cells in the dentate gyrus of adult control and DG-NR1KO mice, which stayed in their home cage or were exposed to a novel environment. Scale bar: 75 μm. ***B***, Density of Arc+/Prox1+ cells in the granule cell layer of adult control and DG-NR1KO mice with or without exposure to the novel environment. ***C***, Normalized density of Arc+/Prox1+ cells in the granule cell layer of adult control and DG-NR1KO mice with or without exposure to the novel environment. Density of each mouse was divided by a mean value of mice without exposure to the novel environment in the same genotype. ***D***, Depth categories divided along the thickness of the granule cell layer. The granule cell layer (GCL) was divided into five 20%-thickness segments, with 0% starting at the border to the hilus and 100% at the border to the molecular layer (ML). ***E***, Density distribution of Arc+/Prox1+ cells along depth; *p < 0.05, **p < 0.01, ***p < 0.005, Tukey’s HSD test.

Confocal imaging was conducted using LSM710 confocal microscope (Zeiss, Germany) equipped with 488-, 543-, and 633-nm laser lines and the ZEN image-acquisition software. For co-localization analysis (BrdU+/Prox1+ and Arc+/Prox1+), a 40× objective lens with oil (NA 1.3) was used. Three images from the granule cell layer from random positions in three different hemispheres/mice were used for BrdU, Prox1 colocalization analysis in Figure 3 and four images from the granule cell layer from random positions in four different mice were used for BrdU, Prox1 colocalization analysis in Figure 6, and Arc, Prox1 colocalization analysis in Figure 7. The density of BrdU+/Prox1+ and BrdU–/Prox1+ were estimated by multiplying the density of BrdU+ cells counted under Axio Scope A1 or Axio Imager M1 microscopes by % of BrdU+ cells which are Prox1+ and Prox1–, measured with the confocal microscope, respectively. For the measurement of the distribution of Arc+/Prox1+ cells in the granule cell layer, only the area that showed the entire depth of the granule cell layer was used from the same confocal images as used for the Arc/Prox1 co-localization counting. Measurements of the granule cell layer area from epifluorescence images and counting of cells from confocal images were performed using ImageJ software (National Institutes of Health).

The section was selected in a way that their anteroposterior level is comparable between mice used in individual analyses. The density was calculated by dividing the number of cells by the volume of the granule cell layer. Our analyses focused on the area covering approximately anterior two thirds of the dentate gyrus, so that the results were from the dorsal dentate gyrus.

### Statistics

Statistical analyses were done using SPSS Statistics (IBM Corp.) and Statistica (StatSoft) software. For independent-sample *t* tests, Levene’s test for equal variance was performed, and depending on the results, a *t* test with (Student’s *t* test) or without (Welch’s *t* test) equal variance assumed was done. All data are presented as mean ± SEM. When we detected significant interaction in two-way ANOVAs or three-factor interaction in three-way ANOVAs, we performed Tukey’s HSD tests. When we detected significant interaction of two factors but not of three factors, we performed simple effects tests using two-way ANOVAs.

## Results

### Reduced neurogenesis and granule cell layer size in the dentate gyrus of adult DG-NR1KO mice

To evaluate neurogenesis in the dentate gyrus of adult control and DG-NR1KO mice, we examined the density of cells expressing Ki67 (a marker for proliferating cells) and DCX (a marker for neuronal progenitor cells and immature neurons). We found that the density of Ki67+ cells is significantly reduced in the subgranular zone of DG-NR1KO mice compared with control (*p* = 0.023, *t*_(11)_ = 2.641, *n* = 6 control and 7 DG-NR1KO mice, independent-sample *t* test, two-tailed; Fig. 3*A*,*B**)*, indicating a reduction in proliferation in DG-NR1KO mice. We also found a significant reduction in the density of DCX+ cells in DG-NR1KO mice compared with control (*p* = 5.6 × 10^−7^, *t*_(11)_ = −10.286, *n* = 6 control and 7 DG-NR1KO mice, independent-sample *t* test, two-tailed; Fig. 3*C*,*D*), indicating a decrease of neuronal progenitor cells and/or immature neurons in the dentate gyrus.

Reduction of DCX+ cells may be exclusively because of reduced proliferation or be partly because of reduced survival of newborn neurons. To examine the latter possibility, we injected BrdU into adult DG-NR1KO and control mice and measured the density of BrdU+/Prox1+ cells 7 or 28 d after injection (Fig. 3*E*). The granule cell marker Prox1 was used to determine the identity of BrdU+ cells as granule cells. Seven days after BrdU injection, the density of BrdU+ cells was significantly lower in DG-NR1KO mice than in controls (*p* = 0.0099, *t*_(14)_ = 2.983, *n* = 7 control and 9 DG-NR1KO mice, respectively, independent-sample *t* test, two-tailed; Fig. 3*F*). Proportion of BrdU+ cells expressing Prox1 was not significantly different between control and DG-NR1KO mice (*p* = 0.657, *t*_(14)_ = −0.454, *n* = 7 and 9 mice, respectively, independent-sample *t* test, two-tailed; Fig. 3*G*), suggesting that neuronal differentiation was not affected. The density of BrdU+/Prox1+ cells showed a tendency of reduction in DG-NR1KO mice, but it was not significant (*p* = 0.070, *t*_(8.385)_ = 2.079, *n* = 7 control and 9 DG-NR1KO mice, respectively, independent-sample *t* test, two-tailed; Fig. 3*H*); 28 d after BrdU injection, both BrdU+ and BrdU+/Prox1+ cell densities were significantly lower in DG-NR1KO mice than in controls (BrdU+: *p* = 3.6 × 10^−4^, *t*_(7.527)_ = 6.116P; BrdU+/Prox1+: *p* = 3.3 × 10^−4^, *t*_(7.367)_ = 6.298, *n* = 8 control and 9 DG-NR1KO mice, independent-sample *t* test, two-tailed; Fig. 3*F*,*H*). Proportion of BrdU+ cells expressing Prox1 was significantly lower in DG-NR1KO mice than in controls (*p* = 5.3 × 10^−5^, *t*_(9.687)_ = 6.834, *n* = 8 control and 9 DG-NR1KO mice, independent-sample *t* test, two-tailed; Fig. 3*G*). Therefore, a proportion of BrdU+ cells that differentiated to neurons were lower in DG-NR1KO mice at 28 d. The density of other cell types (BrdU+/Prox1−) was not significantly different between control and DG-NR1KO mice at either time point (7 d: *p* = 0.366, *t*_(14)_ = 0.934, *n* = 7 and 9 mice, respectively; 28 d: *p* = 0.367, *t*_(15)_ = −0.930, *n* = 8 and 9 mice, respectively, independent-sample *t* tests, two-tailed; Fig. 3*I*). By comparing the density at 7 and 28 d, we calculated the survival rate of new neurons from 7 to 28 d. The survival rate was significantly reduced in DG-NR1KO mice compared with control mice (*p* = 5.0 × 10^−4^, *t*_(7.681)_ = 5.757, *n* = 8 control and 9 DG-NR1KO mice, independent-sample *t* test, two-tailed; Fig. 3*J*).

We also noted that the size of the granule cell layer was smaller in DG-NR1KO mice (Fig. 4*A*). The volume of the granule cell layer (*p* = 4.2 × 10^−4^, *t*_(11)_ = −4.977, *n* = 6 control and 7 DG-NR1KO mice, independent-sample *t* test, two-tailed; Fig. 4*B*), the thickness of the upper and lower blade (*p* = 1.3 × 10^−4^ and 0.0014, *t*_(11)_ = −5.760 and −4.243, respectively, *n* = 6 control and 7 DG-NR1KO mice, independent-sample *t* tests, two-tailed; Fig. 4*C*), and the length of the subgranular zone (*p* = 0.016, *t*_(11)_ = −2.857, *n* = 6 control and 7 DG-NR1KO mice, independent-sample *t* test, two-tailed; Fig. 4*D*) were reduced in DG-NR1KO mice compared with control.

### Reduced neurogenesis and granule cell layer size in juvenile DG-NR1KO mice

It is known that the majority of granule cells in the dentate gyrus are generated during postnatal development (Li and Pleasure, 2005). Thus, the reduced size of the granule cell layer may be caused by reduced neurogenesis during the postnatal development. We therefore examined neurogenesis in postnatal, developing mice. We analyzed Ki67+ cells in the subgranular zone of two-, three-, and four-week-old mice (Fig. 5*A*) and found that the densities of Ki67+ cells were not significantly different between control and DG-NR1KO mice (two weeks: *p* = 0.482, *t*_(11)_ = −0.728, *n* = 5 and 8 mice, respectively, three weeks: *p* = 0.458, *t*_(13)_ = 0.764, *n* = 7 and 8 mice, respectively, four weeks: *p* = 0.610, *t*_(13)_ = 0.522, *n* = 7 and 8 mice, respectively, independent-sample *t* tests, two-tailed; Fig. 5*B*).

The densities of DCX+ cells were not significantly different in the dentate gyrus between control and DG-NR1KO mice at the age of two weeks (*p* = 0.981, *t*_(10)_ = 0.024, *n* = 5 and 7 mice, respectively, independent-sample *t* test, two-tailed; Fig. 5*C*,*D*). But in the three- and four-week-old mice the densities were significantly reduced in the DG-NR1KO mice (three weeks: *p* = 0.0075, *t*_(13)_ = 3.159, *n* = 7 control and 8 DG-NR1KO mice, four weeks: *p* = 0.0048, *t*_(13)_ = 3.394, *n* = 7 control and 8 DG-NR1KO mice, independent-sample *t* tests, two-tailed). Thus, while neurogenesis seems to occur normally initially at the postnatal age of two weeks in DG-NR1KO mice, the number of immature neurons/neuronal progenitor cells starts decreasing between two and three weeks of age. This reduction continues to adulthood in DG-NR1KO mice as shown in Figure 3.

The same developmental pattern of effects was found for the volume of the granule cell layer (Fig. 5*E*,*F*), with no significant difference in two-week-old mice (*p* = 0.500, *t*_(4.515)_ = 0.733, *n* = 5 control and 8 DG-NR1KO mice, independent-sample *t* tests, two-tailed), but significant decreases in three- and four-week-old DG-NR1KO mice compared with control (three weeks: *p* = 0.0070, *t*_(13)_ = 3.197, *n* = 7 control and 8 DG-NR1KO mice, four weeks: *p* = 0.014, *t*_(13)_ = 2.852, *n* = 7 control and 8 DG-NR1KO mice, independent-sample *t* tests, two-tailed).

### Enriched environment exposure increased the survival of newborn neurons in DG-NR1KO mice

Previously it has been shown that exposure to an enriched environment increases the survival of newborn neurons in the dentate gyrus of adult rodents (Kempermann et al., 1997; Tashiro et al., 2007). Exposure to an enriched environment for one week during the second week after neuronal birth has been found to be the most effective time point for this increased survival (Tashiro et al., 2007). To examine whether the NMDA receptors in the dentate gyrus are involved in the effect of enriched environment on the survival of newborn neurons, we injected BrdU on days −1 and 0 and then exposed DG-NR1KO and control mice to an enriched environment or a standard cage with bedding only from day 7 to day 14 (Fig. 6*A*). The enriched environment contained several toys, shelters, tunnels and a running wheel (Fig. 2). Except on days 7–14, the mice were housed in the standard cage. On day 28, we euthanized the mice to quantify the number of BrdU+ and BrdU+/Prox1+ cells (*n* = 7 control-Enriched, 7 control-Standard, 8 DG-NR1KO-Enriched, and 8 DG-NR1KO-Standard mice; Fig. 6*B*). DG-NR1KO mice had significantly lower BrdU+ cell density than control mice while enrichment increases the density similarly in both genotypes (housing: *p* = 0.002, *F*_(1,26)_ = 11.362, genotype: *p* = 4 × 10^−6^, *F*_(1,26)_ = 33.282, housing × genotype: *p* = 0.072, *F*_(1,26)_ = 3.513, two-way ANOVA; Fig. 6*C*). Proportion of BrdU+ cells expressing Prox1 was significantly lower in DG-NR1KO mice than control mice similarly in standard and enriched cages while enrichment did not affect the proportion (housing: *p* = 0.344, *F*_(1,26)_ = 0.928, genotype: *p* = 4.7 × 10^−4^, *F*_(1,26)_ = 16.004, housing × genotype: *p* = 0.967, *F*_(1,26)_ = 0.002, two-way ANOVA; Fig. 6*D*). For BrdU+/Prox1+ cell density (Fig. 6*E*), two-way ANOVA detected significance in the main effect of housing conditions (*p* = 0.002, *F*_(1,26)_ = 11.425) and genotype (*p* = 1 × 10^−6^, *F*_(1,26)_ = 38.776) and interaction between them (*p* = 0.046, *F*_(1,26)_ = 4.410). Exposure to the enriched environment increased the densities in control but not in DG-NR1KO mice (*p* = 0.0047 and 0.785, respectively, df = 26, Tukey’s HSD test) while the densities were lower in DG-NR1KO mice than controls regardless of whether housed in enriched or standard environments (*p* = 1.8 × 10^−4^, and *p* = 0.034, respectively, df = 26, Tukey’s HSD test, two-tailed). However, the percentage increase after exposure to the enriched environment was not significantly different between control and DG-NR1KO mice (housing: *p* = 0.002, *F*_(1,26)_ = 12.336, genotype: *p* = 0.530, *F*_(1,26)_ = 0.404, housing × genotype: *p* = 0.589, *F*_(1,26)_ = 0.299, two-way ANOVA; Fig. 6*F*). Thus, despite overall reduction of neurogenesis in DG-NR1KO mice, exposure to the enriched environment still increased the survival of new neurons in DG-NR1KO mice.

### Abnormalities in novelty-induced Arc gene expression of granule cells in the dentate gyrus of DG-NR1KO mice

Immediate early genes are a group of genes that are expressed in response to neuronal activation. Arc is one of such genes and known to be expressed in rodents after novel environment exploration and learning (Steward et al., 1998; Guzowski et al., 1999, 2001; Czerniawski et al., 2011). The novelty-induced expression of Arc is blocked with an NMDA receptor antagonist (Czerniawski et al., 2011). We examined whether novelty-induced Arc expression requires the NMDA receptors in the dentate gyrus.

For this purpose, we exposed control and DG-NR1KO mice to a novel environment for 10 min and perfusion-fixed them 90 min later. The non-exposed control and DG-NR1KO mice were perfusion-fixed directly from their home cages. We then immunostained the brain sections with anti-Arc and -Prox1 antibodies to detect Arc expressing granule cells and quantified the density of Arc+/Prox1+ cells (*n* = 9 novel environment control, 6 home-cage control, 10 novel environment DG-NR1KO, 10 home-cage DG-NR1KO mice; Fig. 7*A*,*B*). Two-way ANOVA detected significance in the main effect of exposure (home cage vs novel environment, *p* = 5.8 × 10^−9^, *F*_(1,31)_ = 63.087) and genotype (*p* = 9.3 × 10^−13^, *F*_(1,31)_ = 133.291) and interaction between them (*p* = 0.0017, *F*_(1,31)_ = 11.797). Exposure to the novel environment increased the densities in both control and DG-NR1KO mice (*p* = 1.7 × 10^−4^ and 0.008, respectively, df = 31, Tukey’s HSD test) while the densities were lower in DG-NR1KO mice than controls regardless of whether they were exposed to novel environment or not (*p* = 1.7 × 10^−4^ and 2.0 × 10^−4^, respectively, df = 31, Tukey’s HSD test). The percentage increase after exposure to the novel environment was significantly higher in DG-NR1KO mice than in control (exposure: *p* = 7.4 × 10^−8^, *F*_(1,31)_ = 49.031, genotype: *p* = 0.020, *F*_(1,31)_ = 6.028, exposure × genotype: *p* = 0.020, *F*_(1,31)_ = 6.028, two-way ANOVA; *p* = 0.0046, df = 31, Tukey’s HSD test; Fig. 7*C*), while the percentage was significantly increased in both control and DG-NR1KO mice (*p* = 0.027 and 1.7 × 10^−4^, df = 31, Tukey’s HSD test).

We divided the granule cell layer into parallel segments of 20% of the total thickness, and quantified Arc+/Prox1+ cells in each segment (Fig. 7*D*,*E*). Three-way mixed ANOVA detected a significant interaction between depth and genotype (*p* = 8.1 × 10^−5^, *F*_(3.656,113.325)_ = 6.972) but not among depth, genotype and exposure (home cage vs novel environment; *p* = 0.077, *F*_(3.656,113.325)_ = 2.224). These results indicate that, regardless of whether exposed to the novel environment or not, Arc+/Prox1+ cell density showed significantly different changes along depth between control and DG-NR1KO mice. From the subgranular zone (0%) to the molecular layer (100%), control mice show a steady increase in Arc+/Prox1+ cell density up to the middle in the granule cell layer (60–80%), where it reaches a plateau level. In contrast, in DG-NR1KO mice, the density stays low up to the middle of the layer and jumps up at the depth close to the molecular layer (80–100%). Thus, DG-NR1KO mice show an impairment in relative amount of Arc expression along the depth of granule cell layer.

In sum, although DG-NR1KO mice showed a novelty-induced increase in Arc expressing granule cells, we detected impairments in three aspects: (1) overall reduction in Arc+ granule cells regardless of exposure to the novel environment or not; (2) higher percentage increase of Arc+ granule cells after exposure to the novel environment; and (3) abnormal distribution of Arc+ granule cells along the depth of granule cell layer.

### The DG-NR1KO mice display higher tendency to explore the center of an open field during a novelty-suppressed feeding test and show higher fecal counts in an open field test

DG-NR1KO mice have been previously shown to have a deficit in a memory-based context discrimination task (McHugh et al., 2007). Previous studies suggested that adult neurogenesis has a role in anxiety-related behaviors (Jacobs et al., 2000; Santarelli et al., 2003; Surget et al., 2011). Therefore, we examined the behavior of adult, control and DG-NR1KO mice (*n* = 22 and 21 mice, respectively) in the novelty-suppressed feeding test, open field test and elevated plus maze (Santarelli et al., 2003; Walf and Frye, 2007; Seibenhener and Wooten, 2015).

**Figure 8.**
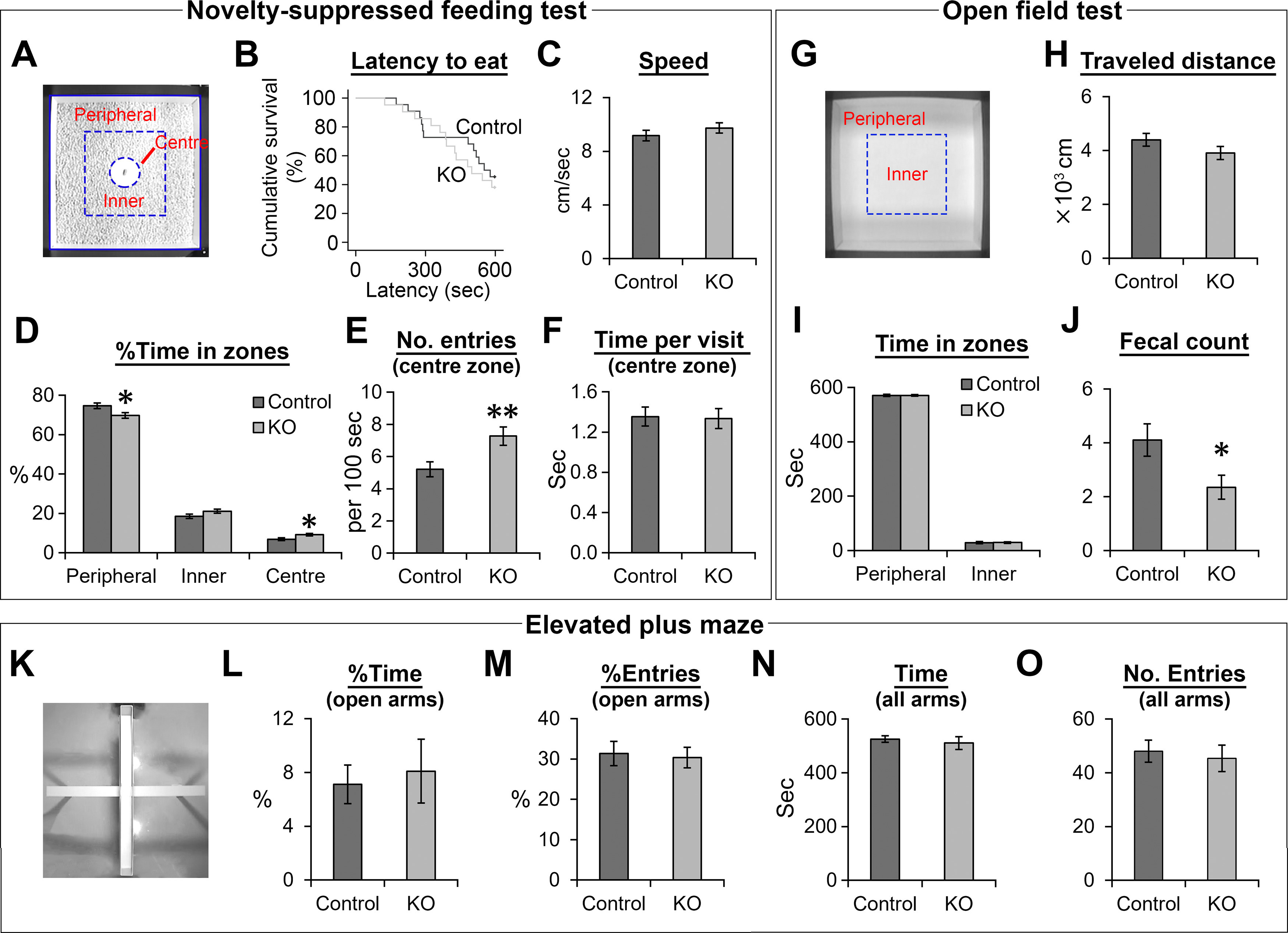
The DG-NR1KO mice display higher tendency to explore the center of an open field during a novelty-suppressed feeding test and show higher fecal counts in an open field test. ***A***, A square open field used in a novelty-suppressed feeding test. Division of the open field into the peripheral, inner and center zone. ***B***, Survival graph for latency to consume food for adult control and DG-NR1KO mice. ***C–F***, Behavioral measurements in the novelty-suppressed feeding test for adult control and DG-NR1KO mice. ***G***, A square open field used in an open field test. Division of the square open field into the peripheral and inner zone. ***H–J***, Behavioral measurements in the open field test for adult control and DG-NR1KO mice. ***K***, An elevated plus maze. ***L–O***, Behavioral measurements in the elevated plus maze for adult control and DG-NR1KO mice; *p < 0.05, **p < 0.01, independent-sample *t* test, two tailed.

After 1-d food deprivation, the mice were exposed to an open field (Fig. 8*A*), which was novel to the mice, with a food pellet located in the center. While mice naturally have a tendency to avoid open unprotected places, hunger drives them to move into the center of the open field to consume the food. The latency to consume the food is often considered to reflect the anxiety level of mice. We did not find a significant difference either in the latency to food consumption (*p* = 0.587, χ^2^(1) = 0.295, Kaplan–Meier survival analysis, log rank test; Fig. 8*B*) or overall speed (*p* = 0.304, *t*_(41)_ = −1.041, independent-sample *t* test, two-tailed; Fig. 8*C*). We performed additional analyses on detailed behavioral performance as described before (Åmellem et al., 2017). The DG-NR1KO mice spent significantly smaller and larger percentages of time in the peripheral and center zones, respectively (*p* = 0.020 and 0.018, *t*_(41)_ = 2.411 and −2.464, respectively, independent-sample *t* tests, two-tailed; Fig. 8*D*), while the difference in the percentage time spent in the inner zone was not significant (*p* = 0.094, *t*_(41)_ = −1.715, independent-sample *t* tests, two-tailed). The number of entries into the center zone per 100 s was significantly higher in the DG-NR1KO mice (*p* = 0.008, *t*_(41)_ = −2.806, independent-sample *t* tests, two-tailed; Fig. 8*E*), while time spent in the center zone per visit was not (*p* = 0.887, *t*_(41)_ = 0.144, independent-sample *t* tests, two-tailed; Fig. 8*F*). These results indicate that the DG-NR1KO mice explore the center of open fields more extensively in the novelty-suppressed feeding test than control, suggesting that the removal of NR1 gene results in this behavioral impairment.

In the open field test (Fig. 8*G*), we quantified three measures which have been related to the anxiety level of mice, traveled distance, time in peripheral versus inner zone and fecal counts. Larger traveled distance, longer time in peripheral zone and higher fecal counts are considered to be indices for higher anxiety level. We did not find significant difference in traveled distance and time in peripheral and inner zones (*p* = 0.157, 0.996, and 0.996, *t*_(34)_ = −1.445, −0.005, and −0.005, respectively, independent-sample *t* test, two-tailed; Fig. 8*H*,*I*). On the other hand, fecal counts are significantly lower in DG-NR1KO mice than control (*p* = 0.028, *t*_(34)_ = −2.296, independent-sample *t* test, two-tailed; Fig. 8*J*). In the elevated plus maze (Fig. 8*K*), we quantified percentage of time in open arms, percentage of entries to open arms, total time in all arms and total number of entries to all arms. None of them show significant difference between control and DG-NR1KO mice (*p* = 0.728, 0.798, 0.581, 0.679, *t*_(34)_ = −0.352, 0.259, 0.560, 0.419, respectively, independent-sample *t* tests, two-tailed; Fig. 8*L–O*).

### The effect of NR1 gene knock-out did not show clear sex difference

**Figure 9.**
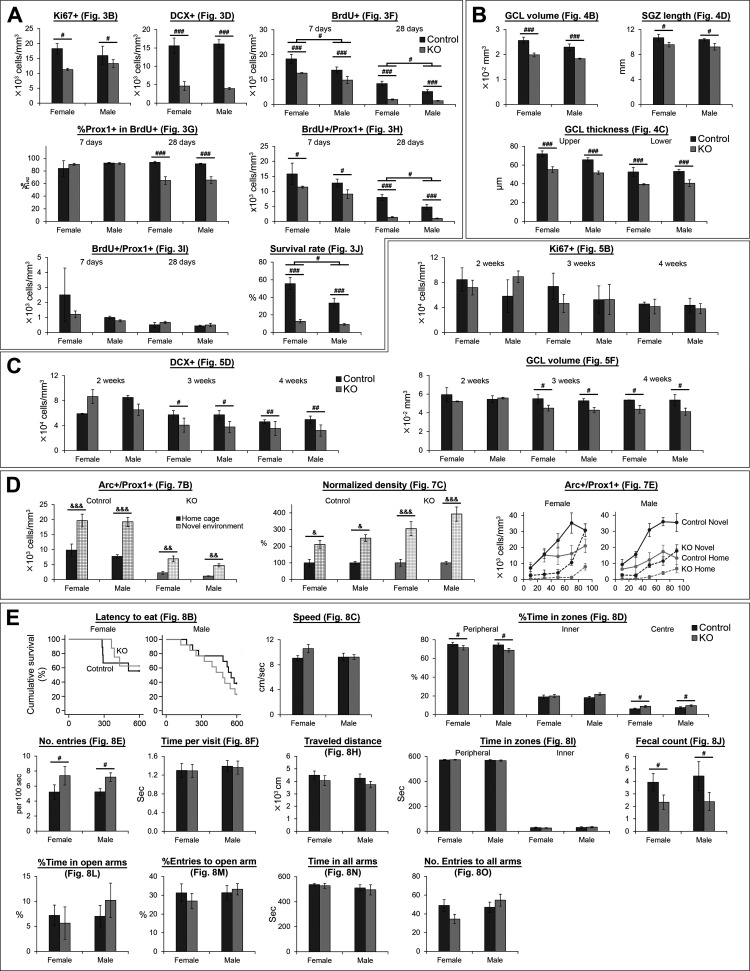
Graphs showing data from female and male mice separately. Data corresponding to Figures 3 (***A***), 4 (***B***), 5 (***C***), 7 (***D***), and 8 (***E***) are shown separately for females and males. Statistical results are summarized in Table 1-Table 14; ^#^*p* < 0.05, ^##^*p* < 0.01, ^###^*p* < 0.005, the main effect of genotype or sex (without significant interaction); ^&^*p* < 0.05, ^&&^*p* < 0.01, ^&&&^*p* < 0.005, Tukey’s HSD test (home cage vs NE including both sexes together and performed each genotype separately; performed because of significant genotype × exposure interaction).

Our original aim did not include the investigation of sex difference. Therefore, although we included both sexes in most experiments except Figure 6 (females only), we did not have a plan to analyze females and males separately. However, in response to reviewers’ requests, we here show the data for females and males separately (Fig. 9). A caveat is that sample size is relatively low for statistical analyses when we divided the data into two sexes, although we believe that the results would still give useful information for future follow-up study. From the graphs in Figure 9, the effect of NR1 gene knock-out seems to be generally consistent between females and males. To support this observation, we performed factorial ANOVA including sex as a between-subject factor (Table 1-Table 14). In most comparisons except six (see the next paragraph for more detail), no significant sex difference was detected as supported by no significance in the main effect of sex or interactions involving sex.

**Table 1:** Two-way ANOVA results for Figure 9*A* **(sex analysis for Fig. 3)**

		Sex	Genotype	Interaction
Fig. 3*B*	*p*	0.932	0.027	0.272
	*F*_(1,9)_	0.008	6.917	1.371
Fig. 3*D*	*p*	0.937	6 × 10^−6^	0.645
	*F*_(1,9)_	0.007	87.273	0.227
Fig. 3*F*(7 d)	*p*	0.010	0.002	0.465
	*F*_(1,12)_	9.215	16.405	0.571
Fig. 3*F* (28 d)	*p*	0.015	5 × 10^−6^	0.069
	*F*_(1,13)_	7.750	55.532	3.920
Fig. 3*G* (7 d)	*p*	0.314	0.567	0.448
	*F*_(1,12)_	1.104	0.346	0.615
Fig. 3*G*(28 d)	*p*	0.861	5.4 × 10^−5^	0.798
	*F*_(1,13)_	0.032	34.581	0.091
Fig. 3*H* (7 d)	*p*	0.130	0.028	0.829
	*F*_(1,12)_	2.637	6.210	0.049
Fig. 3*H*(28 d)	*p*	0.022	4 × 10^−6^	0.064
	*F*_(1,13)_	6.708	57.234	4.113
Fig. 3*I*(7 d)	*p*	0.171	0.276	0.432
	*F*_(1,12)_	2.121	1.301	0.662
Fig. 3*I*(28 d)	*p*	0.360	0.390	0.744
	*F*_(1,13)_	0.889	0.790	0.112
Fig. 3*J*	*p*	0.020	1.2 × 10^−5^	0.081
	*F*_(1,13)_	6.98	46.673	3.575

**Table 2:** Sample size (unit: mice) for Figure 9*A* **(sex analysis for Fig. 3)**

	Genotype	Female	Male
Fig. 3*B,D*	Control	3	3
	KO	3	4
Fig. 3*F–I* (7 d)	Control	3	4
	KO	5	4
Fig. 3*F–I* (28 d)	Control	5	3
	KO	5	4
Fig. 3*J*	Control	5	3
	KO	5	4

**Table 3:** Two-way ANOVA results for Figure 9*B* **(sex analysis for Fig. 4)**

		Sex	Genotype	Interaction
Fig. 4*B*	*p*	0.052	3.7 × 10^−4^	0.524
	*F*_(1,9)_	5.014	30.588	0.440
Fig. 4*C*, upper	*p*	0.093	1.9 × 10^−4^	0.582
	*F*_(1,9)_	3.53	36.882	0.326
Fig. 4*C*, lower	*p*	0.792	0.004	0.928
	*F*_(1,9)_	0.074	14.933	0.009
Fig. 4*D*	*p*	0.523	0.030	0.894
	*F*_(1,9)_	0.441	6.667	0.019

**Table 4:** Sample size (unit: mice) for Figure 9*B* **(sex analysis for Fig. 4)**

Genotype	Female	Male
Control	3	3
KO	3	4

**Table 5:** Two-way ANOVA results for Figure 9*C* **(sex analysis for Fig. 5)**

		Sex	Genotype	Interaction
Fig. 5*B* (week 2)	*p*	0.794	0.595	0.224
	*F*_(1,9)_	0.073	0.304	1.704
Fig. 5*B* (week 3)	*p*	0.729	0.533	0.525
	*F*_(1,11)_	0.126	0.414	0.431
Fig. 5*B* (week 4)	*p*	0.797	0.632	0.947
	*F*_(1,11)_	0.069	0.243	0.005
Fig. 5*D* (week 2)	*p*	0.780	0.673	0.028
	*F*_(1,8)_	0.084	0.192	7.180
Fig. 5*D* (week 3)	*p*	0.813	0.014	0.818
	*F*_(1,11)_	0.058	8.443	0.055
Fig. 5*D* (week 4)	*p*	0.999	0.010	0.463
	*F*_(1,11)_	0.000	9.805	0.579
Fig. 5*F* (week 2)	*p*	0.811	0.306	0.162
	*F*_(1,9)_	0.060	1.176	2.316
Fig. 5*F* (week 3)	*p*	0.533	0.014	0.977
	*F*_(1,11)_	0.415	8.621	0.001
Fig. 5*F* (week 4)	*p*	0.766	0.023	0.790
	*F*_(1,11)_	0.093	6.928	0.074

**Table 6:** Tukey’s HSD test results (control vs DG-NR1KO, two-tailed, df = 8) after significant interaction for Figure 9*C* **(sex analysis for Fig. 5)**

	Female	Male
Fig. 5*D* (week 2)	*p* = 0.253	*p* = 0.363

Four of such exceptions are results corresponding to 7 and 28 d for Figure 3*F* and 28 d for Figure 3*H*,*J*. For these exceptions, the main effects of sex and genotype are significant while their interactions are not significant. Thus, the effects of NR1 gene knock-out occurred similarly in both females and males, although the densities of BrdU+ and BrdU+/Prox1+ cells and survival rate are higher in females regardless of genotype. Another exception is a result corresponding to 7 d in Figure 3*H*. Two-way ANOVA (sex × genotype) showed the main effect of genotype is significant (*p* = 0.028; Tables 1, 2) although the *t* test for the original analysis did not detect this difference as significant (*p* = 0.070). This discrepancy may be because there are variations between females and males (although not significant), which may prevent between-genotype difference in the original analysis from reaching statistical significance in the *t* test when the varied data from two sexes were analyzed without being sorted. The last exception is the analysis corresponding to two weeks old in Figure 5*D*; sex × genotype interaction is significant (Tables 5, 7). However, Tukey’s HSD tests did not reach significance for either female or male (Tables 6, 7). Thus, we did not detect significant difference between control and DG-NR1KO mice in either sex, similarly to the original analysis in Figure 5*D*.

**Table 7:** Sample size (unit: mice) for Figure 9*C* **(sex analysis for Fig. 5)**

	Genotype	Female	Male
Fig. 5*B,F* (week 2)	Control	2	3
	KO	4	4
Fig. 5*D* (week 2)	Control	2	3
	KO	3	4
Fig. 5*B–F* (week 3)	Control	4	3
	KO	4	4
Fig. 5*B–F* (week 4)	Control	3	4
	KO	4	4

**Table 8:** Three-way ANOVA results for Figure 9*D* **(sex analysis for Fig. 7)**

		Sex	Genotype	Exposure	Sex × genotype	Sex × exposure	Genotype × exposure	Sex × genotype × exposure
Fig. 7*B*	*p*	0.136	2.2 × 10^−11^	2.2 × 10^−8^	0.870	0.884	0.002	0.468
	*F*_(1,27)_	2.360	118.582	60.911	0.027	0.022	12.068	0.541
Fig. 7*C*	*p*	0.157	0.011	2.3 × 10^−9^	0.581	0.157	0.011	0.581
	*F*_(1,27)_	2.120	7.548	76.610	0.312	2.120	7.548	0.312

**Table 9:** Four-way mixed ANOVA results, between-subject effects, for Figure 9*D* **(sex analysis for Fig. 7)**

		Sex	Genotype	Exposure	Sex × genotype	Sex × exposure	Genotype × exposure	Sex × genotype × exposure
Fig. 7*E*(between-subjects effects)	*p*	0.730	4.1 × 10^−10^	3.1 × 10^−7^	0.885	0.417	0.145	0.145
	*F*_(1,27)_	0.121	90.569	45.456	0.021	0.680	2.250	2.248

**Table 10:** Four-way mixed ANOVA, within-subject effects, for Figure 9*D* **(sex analysis for Fig. 7)**

		Depth	Depth × sex	Depth × genotype	Depth × exposure	Depth × sex × genotype	Depth × sex × exposure	Depth × genotype × exposure	Depth × sex × genotype × exposure
Fig. 7*E* (within- subjects effects)	*p*	6.7 × 10^−21^	0.138	3.7 × 10^−5^	5.2 × 10^−7^	0.634	0.690	0.117	0.266
	*F*_(4,108)_	41.121	1.781	7.162	10.148	0.641	0.563	1.890	1.323

**Table 11:** Sample size (unit: mice) for Figure 9*D* **(sex analysis for Fig. 7)**

Genotype	Exposure	Female	Male
Control	Home cage	4	2
	Novel	5	4
KO	Home cage	5	5
	Novel	5	5

For survival analysis in Figure 8*B*, we performed survival analysis stratified with sex. We did not detect significant difference between control and DG-NR1KO mice (Fig. 9*E*; Table 12).

**Table 12:** Log rank test result (stratified with sex) for Figure 9*E* **(sex analysis for Fig. 8)**

Fig. 8*B*	*p* = 0.538
	χ^2^(1) = 0.380

**Table 13:** Two-way ANOVA results for Figure 9*E* **(sex analysis for Fig. 8)**

		Sex	Genotype	Interaction
Fig. 8*C*	*p*	0.303	0.188	0.183
	*F*_(1,39)_	1.090	1.799	1.836
Fig. 8*D* (peripheral)	*p*	0.460	0.034	0.619
	*F*_(1,39)_	0.557	4.813	0.251
Fig. 8*D* (inner)	*p*	0.776	0.151	0.397
	*F*_(1,39)_	0.082	2.141	0.734
Fig. 8*D* (left)	*p*	0.247	0.020	0.758
	*F*_(1,39)_	1.381	5.880	0.096
Fig. 8*E*	*p*	0.926	0.010	0.887
	*F*_(1,39)_	0.009	7.322	0.021
Fig. 8*F*	*p*	0.593	0.894	0.930
	*F*_(1,39)_	0.291	0.018	0.008
Fig. 8*H*	*p*	0.432	0.194	0.915
	*F*_(1,32)_	0.633	1.761	0.012
Fig. 8*I* (peripheral)	*p*	0.380	0.983	0.693
	*F*_(1,32)_	0.792	4.7 × 10^−4^	0.158
Fig. 8*I* (inner)	*p*	0.380	0.983	0.693
	*F*_(1,32)_	0.792	4.7 × 10^−4^	0.158
Fig. 8*J*	*p*	0.731	0.030	0.770
	*F*_(1,32)_	0.120	5.187	0.087
Fig. 8*L*	*p*	0.447	0.777	0.410
	*F*_(1,22)_	0.599	0.082	0.704
Fig. 8*M*	*p*	0.431	0.755	0.457
	*F*_(1,22)_	0.644	0.100	0.574
Fig. 8*N*	*p*	0.288	0.646	0.910
	*F*_(1,22)_	1.186	0.216	0.013
Fig. 8*O*	*p*	0.144	0.578	0.081
	*F*_(1,22)_	2.301	0.319	3.335

**Table 14:** Sample size (unit: mice) for Figure 9*E* **(sex analysis for Fig. 8)**

	Genotype	Female	Male
Fig. 8*C–F*	Control	9	13
	KO	8	13
Fig. 8*G–J*	Control	12	7
	KO	9	8
Fig. 8*L–O*	Control	7	6
	KO	6	7

In sum, we did not detect a clear sex difference in the effect of NR1 gene knock-out in any of the analyses in Figure 9.

### Age of mice may affect the effect of NR1 gene knock-out in enrichment-induced survival of new neurons

In most experiments, we used young adult mice from 42 to 60 d old. However, for experiments described in Figures 6, 7, 8*A–F*, we also used more mature adult mice up to 86 d old. Although our original plan did not include the investigation of age difference, we have divided the mice into two groups of <60 and ≥60 d old and analyzed age effect in response to a reviewer’s request. A caveat is that sample size is relatively low for statistical analyses when we divided the data into the two age groups, although we believe that the results would still give useful information for future follow-up study.

**Figure 10.**
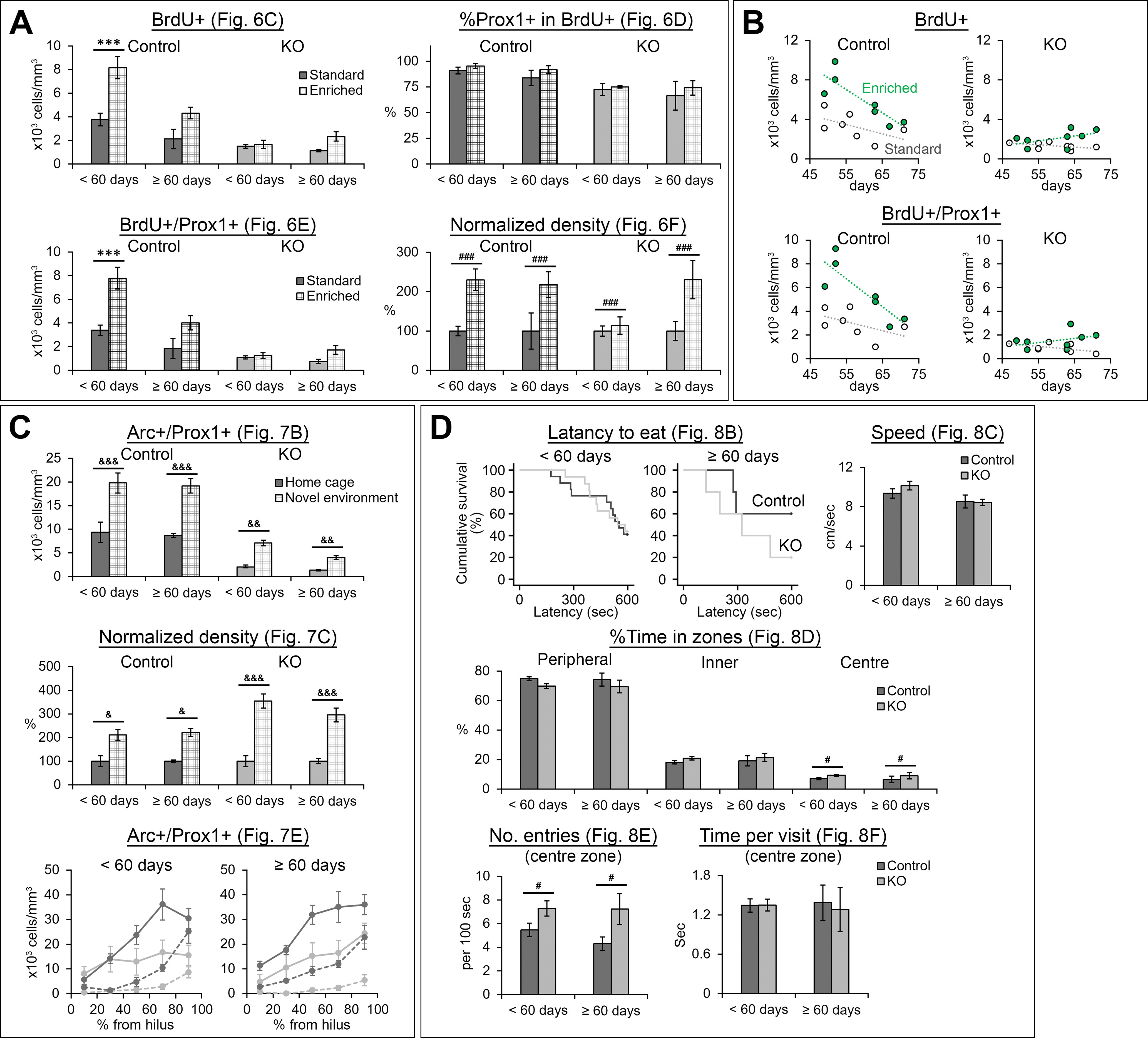
Graphs showing data from <60- and ≥60-d-old mice separately. Data corresponding to Figures 6 (***A***), 7 (***C***), and 8 (***D***) are shown separately for <60- and ≥60-d-old mice. ***B***, Relationship of the densities of BrdU+ and BrdU+/Prox1+ cells with the age of mice at the time of the first BrdU injection. Statistical results are summarized in Table 15-Table 26; ***p < 0.005, Tukey’s HSD test, performed because of significant genotype × housing interaction in two-way ANOVA; ^#^*p* < 0.05, ^###^*p* < 0.005, the main effect of genotype, ^&^*p* < 0.05, ^&&^*p* < 0.01, ^&&&^*p* < 0.005, Tukey’s HSD test (home cage vs novel environment including both age groups together and performed separately for each genotype; performed because of significant genotype × exposure interaction).

The extent of adult neurogenesis is known to be reduced along age. Therefore, as expected, we found that <60-d-old mice have higher densities of BrdU+ and BrdU+/Prox1+ cells (Fig. 10*A*; Table 15-Table 18). Notably, these densities showed significant age × genotype × housing interaction (Tables 15, 18). Then, we performed preplanned comparisons (standard vs enrichment) in four groups (<60-d-old control, ≥60-d-old control, <60-d-old DG-NR1KO, ≥60-d-old DG-NR1KO). The densities of BrdU+ and BrdU+/Prox1+ mice are comparable between standard and enriched conditions in <60-d-old DG-NR1KO mice but the other three group showed significant increase or clear trend of non-significant increase in the densities under enriched conditions (Fig. 10*A*; Tables 16, 18). To further look into this age-dependent differences (Fig. 10*B*), we examined correlation between age and the densities. Enriched control mice show significant, negative correlations between age and BrdU+ or BrdU+/Prox1+ cell density. Standard control and standard DG-NR1KO mice also showed negative correlations, which were not significant probably because of small sample size. On the other hand, enriched DG-NR1KO mice seems to have completely lost the correlations. Because of the limited sample size, this potential age modulation in the effect of NR1 gene knock-out on the densities of BrdU+ and BrdU+/Prox1+ is not fully demonstrated. However, it would be of interest to follow-up this potential difference and elucidate a mechanism behind it.

**Table 15:** Three-way ANOVA results for Figure 10*A,B* **(age analysis for Fig. 6)**

		Age	Genotype	Housing	Age × genotype	Genotype × housing	Age × housing	Age × genotype × housing
Fig. 6*C*	*p*	0.0016	4.4 × 10^−8^	1.5 × 10^−5^	5.7 × 10^−4^	0.0015	0.431	0.035
	*F*_(1,22)_	130.003	66.284	30.354	16.203	13.190	0.643	5.039
Fig. 6*D*	*p*	0.413	0.0021	0.306	0.865	0.906	0.684	0.939
	*F*_(1,22)_	0.696	12.100	1.097	0.030	0.014	0.170	0.006
Fig. 6*E*	*p*	0.0011	1.1 × 10^−8^	1.3 × 10^−5^	6.5 × 10^−4^	7.0 × 10^−4^	0.322	0.037
	*F*_(1,22)_	14.025	78.211	31.159	15.720	15.538	1.028	4.920
Fig. 6*F*	*p*	0.279	0.282	4.0 × 10^−4^	0.185	0.282	0.279	0.185
	*F*_(1,22)_	1.234	1.216	17.361	1.871	1.216	1.234	1.871

**Table 16:** Tukey’s HSD test results (standard vs enrichment, two-tailed, df = 22) after significant interaction for Figure 10*A,B* **(age analysis for Fig. 6)**

	Genotype	<60 d	≥60 d
Fig. 6*C*	Control	*p* = 1.9 × 10^−4^	*p* = 0.182
	KO	*p* = 1.000	*p* = 0.575
Fig. 6*E*	Control	*p* = 1.7 × 10^−4^	*p* = 0.156
	KO	*p* = 1.000	*p* = 0.737

**Table 17:** Age (mean ± SD) for Figure 10*A,B* **(age analysis for Fig. 6)**

	Housing	<60 d	≥60 d
Control	Standard	53.2 ± 4.1	67.0 ± 5.7
	Enrichment	51.0 ± 1.7	66.0 ± 3.8
KO	Standard	53.8 ± 4.7	65.5 ± 3.7
	Enrichment	51.0 ± 1.7	65.6 ± 3.4

**Table 18:** Sample size (unit: mice) for Figure 10*A,B* **(age analysis for Fig. 6)**

	Housing	<60 d	≥60 d
Control	Standard	5	2
	Enrichment	3	4
KO	Standard	4	4
	Enrichment	3	5

**Table 19:** Pearson correlation between age and density for Figure 10*A,B* **(age analysis for Fig. 6)**

	BrdU+		BrdU+/Prox1+	
	Standard	Enriched	Standard	Enriched
Control	*p* = 0.202	*p* = 0.015	*p* = 0.250	*p* = 0.017
	*r* = −0.548	*r* = −0.851	*r* = −0.503	*r* = −0.844
KO	*p* = 0.188	*p* = 0.199	*p* = 0.118	*p* = 0.323
	*r* = −0.518	*r* = 0.507	*r* = −0.597	*r* = 0.403

**Table 20:** Three-way ANOVA results for Figure 10*C* **(age analysis for Fig. 7)**

		Age	Genotype	Exposure	Age × genotype	Genotype × exposure	Age × exposure	Age × genotype × exposure
Fig. 7*B*	*p*	0.197	1.1 × 10^−11^	4.0 × 10^−8^	0.536	0.0017	0.541	0.515
	*F*_(1,27)_	1.752	125.968	57.115	0.394	12.081	0.383	0.435
Fig. 7*C*	*p*	0.519	0.0072	1.0 × 10^−9^	0.367	0.0072	0.519	0.367
	*F*_(1,27)_	0.428	8.444	82.771	0.841	8.444	0.428	0.841

**Table 21:** Four-way mixed ANOVA results, between-subject effects, for Figure 10*C* **(age analysis for Fig. 7)**

		Age	Genotype	Exposure	Age × genotype	Age × exposure	Genotype × exposure	Age × genotype × exposure
Fig. 7*E*(between- subjects effects)	*p*	0.295	1.9 × 10^−10^	3.8 × 10^−7^	0.384	0.259	0.246	0.847
	*F*_(1,27)_	1.141	97.378	44.365	0.784	1.332	1.406	0.038

**Table 22:** Four-way mixed ANOVA results, within-subject effects, for Figure 10*C* **(age analysis for Fig. 7)**

		Depth	Depth × age	Depth × genotype	Depth × exposure	Depth × age × genotype	Depth × age × exposure	Depth × genotype × exposure	Depth × age × genotype × exposure
Fig. 7*E* (within- subjects effects)	*p*	3.4 × 10^−20^	0.735	2.0 × 10^−4^	1.0 × 10^−5^	0.295	0.697	0.101	0.822
	*F*_(4,108)_	39.068	0.501	6.039	8.041	1.248	0.553	1.992	0.380

**Table 23:** Sample size (unit: mice) for Figure 10*C* **(age analysis for Fig. 7)**

	Exposure	<60 d	≥60 d
Control	Home cage	4	2
	Novel	5	4
KO	Home cage	6	4
	Novel	6	4

**Table 24:** Log rank test results (stratified with age) for Figure 10*D* **(age analysis for** Fig. 8*B–F***)**

Fig. 8*B*	*p* = 0.660
	χ^2^(1) = 0.194

**Table 25:** Two-way ANOVA results of Figure 10*D* **(age analysis for** Fig. 8*B–F***)**

		Age	Genotype	Interaction
Fig. 8*C*	*p*	0.052	0.582	0.489
	*F*_(1,39)_	4.022	0.308	0.487
Fig. 8*D*(peripheral)	*p*	0.862	0.058	0.951
	*F*_(1,39)_	0.031	3.815	0.004
Fig. 8*D* (inner)	*p*	0.669	0.186	0.900
	*F*_(1,39)_	0.185	1.808	0.016
Fig. 8*D* (left)	*p*	0.750	0.044	0.941
	*F*_(1,39)_	0.103	4.312	0.005
Fig. 8*E*	*p*	0.495	0.010	0.526
	*F*_(1,39)_	0.474	7.23	0.410
Fig. 8*F*	*p*	0.940	0.770	0.742
	*F*_(1,39)_	0.006	0.087	0.110

**Table 26:** Sample size (unit: mice) for Figure 10*D* **(age analysis for** Fig. 8*B–F***)**

	<60 d	≥60 d
Control	17	5
KO	16	5

None of other measurements corresponding to Figures 6*D*,*F*, 7, 8*B–F* (Fig. 10*A*,*C*,*D*) showed any notable age effect (main effects of age or interactions involving age; Table 15-Table 26).

## Discussion

In this study, we have made three major findings. First, DG-NR1KO mice show impairment in neurogenesis during postnatal development and adulthood (Figs. 3, 5*A–D*). Second, DG-NR1KO mice have smaller size of the granule cell layer (Fig. 4), which becomes apparent during postnatal development (Fig. 5*E*,*F*). Third, DG-NR1KO mice have a functional deficit, which appears as increased exploration of the center of the open field during the novelty-suppressed feeding test and reduced fecal counts in the open field test (Fig. 8). Considering the fact that the NR1 gene is essential for the formation of functional NMDA receptors, these findings suggest that the NMDA receptors are essential for normal development and function of the dentate gyrus. McHugh et al. (2007) showed that the Cre-mediated removal of NR1 genes is specific to granule cells in the dentate gyrus. Based on this finding, our following discussion focuses on a role of the NMDA receptors in granule cells. However, a technical caveat for region-specific or cell type-specific knock-out mice is that one cannot completely exclude the possibility that the observed phenotype may be caused by gene knock-out in other regions or cell types than the regions or cell type of interest. We suggest readers to read our discussion while keeping this caveat in mind.

### The NMDA receptors in granule cells support the normal development of the dentate gyrus

One of our major findings is that the size of the granule cell layer was smaller in the dorsal dentate gyrus of DG-NR1KO mice compared with control mice (Fig. 4), suggesting that the NR1 gene in granule cells is required for the normal development of the granule cell layer. This finding is consistent with a study by Bannerman and colleagues, which reported a reduced size of the dentate gyrus in mice lacking the NR1 gene in the hippocampus but not specifically in granule cells (Bannerman et al., 2012). In contrast to our finding, McHugh et al. (2007) did not report abnormality in the size of granule cell layer. However, considering high variability of granule cell layer size among coronal sections along the anteroposterior axis, relatively small reduction in size (∼20%; Fig. 4) can be easily missed unless one systematically examines many sections and quantifies the volume as we did. We further found that this reduced size becomes apparent as early as at the postnatal age of three weeks (Fig. 5*E*,*F*), which demonstrates the developmental origin of this deficit.

We examined neurogenesis during the first few weeks of postnatal period, when the majority of granule cells in the dentate gyrus are generated (Encinas et al., 2013). We did not find any change in the number of Ki67+ cells up to the postnatal age of four weeks (Fig. 5*B*), which suggests that cell proliferation is intact during postnatal development. In contrast, we detected a reduction of DCX+ cells starting at the postnatal age of three weeks (Fig. 5*D*), indicating that neurogenesis is impaired in the postnatal, developing dentate gyrus of DG-NR1KO mice. Because cell proliferation is not affected, this reduction is likely through a later process of neurogenesis such as neuronal survival. The reduction appears at the same timing as reduced size of granule cell layer (Fig. 5*E*,*F*), suggesting that impaired neurogenesis contributes to the reduction in the size of granule cell layer.

### DG-NR1KO mice show impaired adult neurogenesis in the dentate gyrus

In DG-NR1KO mice, we found impairment in adult neurogenesis in the dorsal dentate gyrus. This impairment was shown as reduction in Ki67+, DCX+, BrdU+, and BrdU+/Prox1+ cells (Figs. 3*A–F*,*H*, 9*A*). Overall, the number of approximately four-weeks-old, newly-generated neurons are reduced by >80% in DG-NR1KO mice (Fig. 3*H*). The reduced ratio of BrdU+/Prox1+ cells at 28 d to 7 d (Fig. 3*J*) indicates that the survival of newly-generated neurons is compromised. The result suggests that the NMDA receptors in the granule cells support the survival of new neurons in the adult dentate gyrus. The finding is consistent with previous studies which showed that the NMDA receptor-dependent long-term potentiation increases the survival of newborn neurons in the dentate gyrus (Bruel-Jungerman et al., 2006) and that the NMDA receptors on immature neurons regulate their own survival (Tashiro et al., 2006b).

A reduction of Ki67+ cells indicates a deficit in cell proliferation in adult DG-NR1KO mice, which was not observed in the postnatal, developing dentate gyrus. Considering that the impairment in neurogenesis (a reduction of DCX+ cells) and granule cell layer size precedes the reduction of Ki67+ cells, the effect on cell proliferation may be secondary to the abnormal development of the dentate gyrus. In addition, this possibility is also supported by the previous finding that the Pomc promoter initiates transcription in newborn neurons at some time between 3 and 11 d of neuronal age but not in progenitor cells (Overstreet et al., 2004). Therefore, NR1 gene knock-out is unlikely occur in proliferating cells.

This reduction of cell proliferation in the dentate gyrus of adult DG-NR1KO mice is in contrast to the previous studies which suggested NMDA receptor activation inhibits cell proliferation (Cameron et al., 1995; Gould and Cameron, 1997). These studies found that the systemic injection of NMDA receptor agonist and antagonists, respectively, decreases and increase cell proliferation in the adult dentate gyrus. This discrepancy may be because systemic injection blocks NMDA receptor function in the brain regions other than the dentate gyrus while NR1 gene removal in DG-NR1KO mice is specific to the dentate gyrus. Alternatively, it may be because of acute activation/blockade by the injection of an agonist/antagonist compared with chronic blockade starting from postnatal development in DG-NR1KO mice.

We found a reduced proportion of BrdU+ cells expressing Prox1 in DG-NR1KO mice at 28 d, but not 7 d, after BrdU injection (Fig. 3*G*). The proportion reached ∼90% 7 d after BrdU injection and did not show difference between control and DG-NR1KO mice. Until 28 d, the proportion stayed at ∼90% in control mice but reduced to ∼60% in DG-NR1KO mice. The reduced proportion of BrdU+ cells expressing a neuronal marker is often interpreted as reduced tendency to neuronal differentiation. However, we believe that this reduction was caused by selective reduction in the survival of BrdU+/Prox1+ cells in DG-NR1KO mice in combination with the intact survival of BrdU+/Prox1– cells. However, the reviewer believes that this is partly caused by reduced neuronal differentiation. We believe that this is unlikely or, if any, makes only a small contribution because of the following reasons. First, by 7 d after BrdU injection, ∼90% of BrdU+ cells had already committed to neuronal fate (Prox1+) and no further increase happened from 7 to 28 d after BrdU injection in control mice, suggesting that no (or only small) further neuronal differentiation normally occurs after 7 d. Second, the proportion of BrdU+ cells expressing Prox1+ reduced from ∼90% to ∼60%. If neuronal differentiation is a major cause of this reduction, de-differentiation from neuronal fate (losing Prox1 expression) needs to occur. Although we believe that reduced neuronal differentiation in DG-NR1KO mice is unlikely, we agree with the reviewer that our result does not completely exclude this possibility and that reduced neuronal differentiation or increased de-differentiation may have contributed to reduced neurogenesis in DG-NR1KO mice.

Exposure to an enriched environment has previously been shown to increase the survival of newborn neurons in the adult dentate gyrus (Kempermann et al., 1997; Tashiro et al., 2007), and our results confirm these findings (Fig. 6). Although the overall level of BrdU+/Prox1+ cells were lower in DG-NR1KO mice compared with control (Fig. 6*E*), the percentage increase of BrdU+/Prox1+ cells after exposure to the enriched environment did not differ significantly between the two genotypes (Fig. 6*F*). Therefore, the NMDA receptors in granule cells are not essential for enrichment-induced increase in survival of new neurons, although we cannot exclude the possibility that there is a sub-component of a survival-inducing effect which is dependent on the NMDA receptors.

### The NMDA receptors in granule cells are required for normal function of the dentate gyrus

The immediate early gene Arc has been shown to be involved in synaptic plasticity in the hippocampus and in the consolidation of long-term memory (Guzowski et al., 2000; Plath et al., 2006), and it is known that Arc expression is induced by novel environment exploration, learning, and high-frequency stimulation (Steward et al., 1998; Guzowski et al., 1999, 2001), and is dependent on NMDA receptor function (Czerniawski et al., 2011).

We found a significantly lower number of Arc+/Prox1+ cells in the DG-NR1KO compared with the control mice (Fig. 7*B*), which confirms that Arc expression is dependent on functional NMDA receptors in granule cells. This reduction is regardless of whether the mice were exposed to the novel environment or not. In addition, we found that, on exposure to the novel environment, Arc+/Prox1+ cells still increase in DG-NR1KO mice, and in terms of percentage changes, the novelty-induced increase is higher in DG-NR1KO mice than control (Fig. 7*C*). These findings indicate that, although Arc expression is dependent on the NMDA receptors, at least some components of a novelty-induced increase in Arc expression are not dependent on the NMDA receptors; there may co-exist mechanisms dependent on and independent of the NMDA receptors.

Another observation we made was a between-genotype difference in the distribution of Arc+/Prox1+ cells along the depth of the granule cell layer (Fig. 7*E*). Both control and DG-NR1KO mice had higher numbers of Arc+/Prox1+ cells toward the outer part of the granule cell layer. However, with or without exposure to the novel environment, control mice increased the number of Arc+/Prox1+ cells starting from the inner half of the layer. In contrast, DG-NR1KO mice kept the numbers of Arc+/Prox1+ cells low in the inner half and showed higher number of Arc+/Prox1+ cells mainly in the most outer part (80–100%) of the layer. This finding suggests that functioning of the dentate gyrus is compromised in DG-NR1KO mice.

Overall, these three observed abnormalities in Arc expression, (1) overall reduction in Arc+ granule cells; (2) an enhanced, novelty-induced increase in terms of percentage changes; and (3) abnormal distribution across the depth of granule cell layer, suggest that the function of the dentate gyrus, which is mediated by the Arc gene, may be compromised.

The novelty-suppressed feeding test is a conflict paradigm in which a food-deprived mouse has to choose between going into an open space to get food or stay close to the walls without access to the food. The latency until the mouse starts to eat the food has been considered as a measure of anxiety level of the mouse. Santarelli and colleagues found that the blockade of neurogenesis abolished the behavioral effect of fluoxetine in the novelty-suppressed feeding test (Santarelli et al., 2003). Adult neurogenesis has been linked to anxiety and mood disorders (Gould et al., 1997; Jacobs et al., 2000). Studies have found increased anxiety after a mild stressor (Snyder et al., 2011) or increased anxiety without a stressor (Revest et al., 2009) after ablation of adult neurogenesis. While we did not find a significant difference in the latency to consume food, we found multiple measurements pointing to an increased tendency for DG-NR1KO mice to explore the center of the open field, which is also often considered as a measure reflecting animal’s anxiety level, for example, in an open field test (Seibenhener and Wooten, 2015). In the open field test, we did not find similar tendency to spend more time in the inner part of the open field. However, we detected a significant reduction in fecal counts during the open field test, which has been considered as an index of lower anxiety level (Seibenhener and Wooten, 2015). From these mixed results, one may be inclined to interpret it as an indication that DG-NR1KO mice exhibit lower anxiety level. However, we are hesitant to conclude that the loss of the NMDA receptors in granule cells results in an anxiety-related phenotype. On the other hand, it is safe to say that DG-NR1KO mice have behavioral abnormality which is not necessarily based on compromised memory mechanisms which has been previously characterized (McHugh et al., 2007). This knowledge would be important to interpret their behavioral phenotypes involving spatial exploration and/or emotion.

Our work has contributed to the understanding of the role of the NMDA receptors in the normal development and adult neurogenesis of the dentate gyrus, and that the loss of the NMDA receptors during brain maturation can contribute to permanent alterations in brain function. Future work is required to reveal the complex contribution of the NMDA receptors to brain development and function in different brain areas.

## Synthesis

Reviewing Editor: Aniko Korosi, University of Amsterdam Decisions are customarily a result of the Reviewing Editor and the peer reviewers coming together and discussing their recommendations until a consensus is reached. When revisions are invited, a fact-based synthesis statement explaining their decision and outlining what is needed to prepare a revision will be listed below. The following reviewer(s) agreed to reveal their identity: Christoph Anacker, Djoher Nora Abrous.

Reviewer 1

In this paper, the authors conduct a careful and interesting characterization of the role of NR1 NMDA receptors for adult hippocampal neurogenesis and dentate gyrus development. Using a POMC-Cre transgenic mouse line, the authors delete NR1 specifically from granule cells of the dentate gyrus and find that granule cell specific deletion of NR1A decreases cell proliferation, neuronal survival, granule cell layer volume, and expression of the immediate early gene Arc in Prox1+ granule cells. The authors also find that this deficit in neurogenesis and immediate early gene expression is partially counteracted by environmental enrichment. The findings are interesting and provide new insight into how NMDA receptors contribute to adult hippocampal neurogenesis and dentate gyrus development. I hope that the following comments will be helpful to further improve the current manuscript.

1. The POMC-Cre mouse line that was used in this study is a leaky mouse line, and germline recombination occurs frequently when the Cre allele is passed through the female germline. Did the authors ensure in their heterozygote crossings that Cre is always passed through the male germline? It would be helpful to see some confirmation that NR1A is indeed specifically deleted from granule cells and not in the entire brain (e.g. using a floxed-GFP mouse or immunohistochemistry for NR1A). Confirming that the knockdown is specific to the dentate will be important, as the authors emphasize the cell-autonomous effect of NR1A in granule cells.

2. The POMC promoter utilized in this transgenic mouse line is also expressed in the hypothalamus. Did the authors check if NR1A was also deleted in POMC neurons in the hypothalamus? Could it be possible that NR1A-dependent changes in the activity of hypothalamic neurons may influence HPA axis activity and corticosterone levels, which could in turn contribute indirectly to many of the interesting effects on neurogenesis seen in this study?

3. The images in Figure 2-6 show the dorsal portion of the dentate gyrus. Were similar effects on neurogenesis found in the ventral dentate gyrus?

4. The behavioral data in the NSF test is difficult to interpret. The authors do not find an effect on feeding latency, but a small increase in the time spent in the center of the NSF arena. Did the authors control for differences in home cage food consumption or home cage feeding latency? I would recommend to either include a more comprehensive behavioral assessment in complementary tasks (e.g. open field, elevated plus maze) or to remove the behavior data in Figure 7 altogether, as on its own, it is very difficult to interpret.

5. I would recommend to move the image of the environmental enrichment cage to the Supplementary data, instead of showing it as Figure 1.

Reviewer 2

In this manuscript the authors investigated the role of NMDA receptors in adult hippocampal neurogenesis using a transgenic approach. It is an interesting study but I have several recommendations to improve the manuscript.

Paper organization

• Depending on the title and sequence of the introduction, results on the DG under development should be presented first. However, given the paucity of data collected on developmental neurogenesis, and the fact that this paper does not systematically compare adult and developmental neurogenesis, the title should be changed as well as the introduction. Developmental results should be introduced more simply. Indeed in adult mice there is a decrease in volume that should result from the mutation of NR1 during development.

Methods:

• At 6 weeks the mice are not adults so results from 6 and 12 weeks old mice should be separated.

• Results obtained for male and female mice should be presented separately.

• Were male and female mice exposed to the EE?

• The critical values and ddl for the statistical analysis should be provided

• Confocal analysis has not been used to determine the phenotype of BrdU cells. This caveat should be discussed.

Results

• The model need to be better described if not published previously.

• I am not convinced by the lack of difference in Brdu-prox1 cells 7 days after the injection (Fig 2F). They are at least 2 explanations: 1) inter-individual differences due to sex difference and or low number of animals or to difference in the age of the animals (6 to 12?), 2) results are biased by differences in neuronal differentiation. Can the data of BrdU without prox1 be provided?

• Why is there a difference in the volume of control mice in Fig3B and Fig 4F?

Discussion

• as the results obtained are different from those of Mc Hugh et al, a comparison of the two genetic models is essential

• The authors concluded that the NR1 gene in granule cells is required for the normal development of the granule. This is incorrect. Dentate granule neurons generated during embryogenesis and the first 2 postnatal weeks do not seem to be impacted by genetic NR1manipulation. But see Gould & Cameron 1997

• Line 349: here (and not in the intro) discuss McHugh et al. !

• 353: the term postnatal neurogenesis is confusing. Adult neurogenesis also occurs postnatally

• Paragraph line 385. The data of Gould&Cameron 1997 deserve to be included in the discussion.

• The behavioural test is not clearly justified.

• The results seem to indicate a deficit in anxiety like-responses. Another would be welcome to confirm this data (Plus maze)

## Author Response

1

Reviewer 1

In this paper, the authors conduct a careful and interesting characterization of the

role of NR1 NMDA receptors for adult hippocampal neurogenesis and dentate gyrus

development. Using a POMC-Cre transgenic mouse line, the authors delete NR1

specifically from granule cells of the dentate gyrus and find that granule cell specific

deletion of NR1A decreases cell proliferation, neuronal survival, granule cell layer

volume, and expression of the immediate early gene Arc in Prox1+ granule cells. The

authors also find that this deficit in neurogenesis and immediate early gene

expression is partially counteracted by environmental enrichment. The findings are

interesting and provide new insight into how NMDA receptors contribute to adult

hippocampal neurogenesis and dentate gyrus development. I hope that the following

comments will be helpful to further improve the current manuscript.

1. The POMC-Cre mouse line that was used in this study is a leaky mouse line, and

germline recombination occurs frequently when the Cre allele is passed through the

female germline. Did the authors ensure in their heterozygote crossings that Cre is

always passed through the male germline?

This germline recombination issue can be found in the description of the POMC-CRE

mouse line in the Jackson Laboratory website as below.

"According to the donating investigator, passing the cre through the female germline

can result in a high percentage of offspring that carry the recombined allele in all of

their tissues. Such “leaky” Cre expression is reported in male POMC-cre mice also,

but at a lower frequency (< 5%).” (https://www.jax.org/strain/010714)

Considering this information, we have extensively performed PCR genotyping for the

recombined allele as described in Materials and Methods section in our original

manuscript. We confirmed the information provided by the donating investigator that

germline recombination occurs in both male and female Cre carriers but at higher

frequency in females. We routinely checked the presence of recombined alleles in

genome DNA. In the course of our study, we found several Cre-negative mice with

germline recombined alleles and they were removed from our analyses.

It would be helpful to see some confirmation that NR1A is indeed specifically deleted

from granule cells and not in the entire brain (e.g. using a floxed-GFP mouse or

immunohistochemistry for NR1A). Confirming that the knockdown is specific to the

dentate will be important, as the authors emphasize the cell-autonomous effect of

NR1A in granule cells.

We regret that our description of DG-NR1KO mice was not clear. We would like to

make sure with the reviewer that we purchased the exactly same POMC-CRE

(B6.FVB-Tg(Pomc-cre)1Lowl/J) and floxed NR1 (B6.129S4-Grin1tm2Stl/J) mouse

lines as McHugh et al. (2007) used and re-generated DG-NR1KO mice. Therefore,

our line is same as McHugh used. To avoid the misunderstanding, we revised Ln 59-

60 and Ln 72-73.

McHugh et al. (2007) crossed the same POMC-CRE mouse line with a mouse line

with cre-dependent expression of lacZ, and found lac Z expression occurs specifically

in the granule cell layer of the dentate gyrus, the lateral habenular nucleus and the 2

arcuate nucleus of the hypothalamus as shown below (Fig. 2A in McHugh et al.,

2007).

However, in situ hybridization analysis in DG-NR1KO mice showed reduction of NR1

transcripts only in the dentate gyrus but not in other brain regions including the lateral

habenular nucleus and the arcuate nucleus as shown below (Fig. 2A-D, K-N, in

McHugh et al., 2007).

In this way, specificity of Cre-mediated NR1 gene knockout has been validated in

DG-NR1KO mice.

Nonetheless, in general, even with careful examination such as McHugh et al. (2007)

did, leaky Cre expression and gene removal in unintended brain regions are always a

potential caveat in the use of region-specific conditional gene knockout mice.

Therefore, we added description about this potential caveat in Ln 483-489.3

Ln 483-489 - “McHugh et al. (2007) showed that the Cre-mediated removal of NR1

genes is specific to granule cells in the dentate gyrus. Based on this finding, our

following discussion focuses on a role of the NMDA receptors in granule cells.

However, a technical caveat for region- or cell-type-specific knockout mice is that one

cannot completely exclude the possibility that the observed phenotype may be

caused by gene knockout in other regions or cell types than the regions or cell type

of interest. We suggest readers to read our discussion while keeping this caveat in

mind.”

2. The POMC promoter utilized in this transgenic mouse line is also expressed in the

hypothalamus. Did the authors check if NR1A was also deleted in POMC neurons in

the hypothalamus? Could it be possible that NR1A-dependent changes in the activity

of hypothalamic neurons may influence HPA axis activity and corticosterone levels,

which could in turn contribute indirectly to many of the interesting effects on

neurogenesis seen in this study?

As we explained in the response to comment #1, McHugh et al. (2007) performed in

situ hybridization analysis and did not detect reduction in NR1 transcript in the

arcuate nucleus of the hypothalamus, where POMC neurons are located.

Nonetheless, even with careful examination such as McHugh et al. (2007) did, leaky

Cre expression and gene removal in unintended brain regions are always a potential

caveat in the use of region-specific conditional gene knockout mice. Therefore, we

add description about this potential caveat in Ln 483-489.

3. The images in Figure 2-6 show the dorsal portion of the dentate gyrus. Were

similar effects on neurogenesis found in the ventral dentate gyrus?

We harvested coronal brain sections containing roughly rostral two thirds of the

dentate gyrus; therefore, the result presented in this study is mostly from the dorsal,

but not ventral, part of the dentate gyrus. We made this clear by adding descriptions

in Ln 154-156 and 201-202 in Materials and Methods and also specifying it in Ln 8 in

abstract, and in Ln 492 and 517 in discussion.

4. The behavioral data in the NSF test is difficult to interpret. The authors do not find

an effect on feeding latency, but a small increase in the time spent in the center of

the NSF arena. Did the authors control for differences in home cage food

consumption or home cage feeding latency? I would recommend to either include a

more comprehensive behavioral assessment in complementary tasks (e.g. open field,

elevated plus maze) or to remove the behavior data in Figure 7 altogether, as on its

own, it is very difficult to interpret.

If the reviewer means that it is difficult to interpret the result as an anxiety phenotype,

we agree with it. However, we believe that it is easy and safe to interpret the result as

a behavioural abnormality which is not based on compromised memory mechanisms,

as we wrote in Ln 450-453 in the original manuscript (Ln 605-608 in the new

manuscript). We believe that it is not a good practice to remove and ignore this kind

of results. We did not measure home cage food consumption or feeding latency.4

In response to the reviewer’s recommendation, we added the results from an open

field test (Figure 7G-J) and an elevated plus maze (Figure 7K-O). We did not find

significant difference in travelled distance and time spent in peripheral/inner zones in

the open field test and %Time in and %Entries to open arms in the elevated plus

maze. However, DG-NR1KO mice showed significant decrease in fecal counts during

the open field test, which has been used for an indication of anxiety level

(Seibenhener et al., J Vis Exp, 96: e52434, 2015). Lower fecal counts has been

considered to reflect lower anxiety level. Thus, from the three anxiety-related tasks,

two measures (%time in peripheral/centre zone in novelty-suppressed feeding test

and fecal counts in open field test) suggest lower anxiety level in DG-NR1KO mice

while other measures used as indices for anxiety level did not show difference.

Therefore, although we are still hesitant to conclude an effect on anxiety level from

these mixed results, the additional results have made it more convincing that there is

some non-memory-related behavioural abnormality in DG-NR1KO mice.

Figure 7G-O

5. I would recommend to move the image of the environmental enrichment cage to

the Supplementary data, instead of showing it as Figure 1.

We believe that this is a matter of style preference, and there is no right or wrong

styles for this. This is why we keep our original style. Also, eNeuro does not accept

supplementary material, except (extended) data. We hope the reviewer accepts our

personal preference.5

Reviewer 2

In this manuscript the authors investigated the role of NMDA receptors in adult

hippocampal neurogenesis using a transgenic approach. It is an interesting study but

I have several recommendations to improve the manuscript.

Paper organization

• Depending on the title and sequence of the introduction, results on the DG under

development should be presented first. However, given the paucity of data collected

on developmental neurogenesis, and the fact that this paper does not systematically

compare adult and developmental neurogenesis, the title should be changed as well

as the introduction. Developmental results should be introduced more simply. Indeed

in adult mice there is a decrease in volume that should result from the mutation of

NR1 during development.

We thought that reduced DCX+ cells and granule cell layer size are sufficient to claim

that NMDA receptor knockout impaired neurogenesis during postnatal development.

However, we understand the point the reviewer made and now feel that our results

may not be weak for claiming a role of NMDA receptor in neurogenesis in juvenile

brains. On the other hand, as the reviewer pointed out, the reduced size of granule

cell layer is a clear indication of a role of NMDA receptor in normal development of

the dentate gyrus. Therefore, we have revised the title to “Role of NMDA receptors in

adult neurogenesis and normal development of the dentate gyrus.”

In addition, now we feel that the original introduction does not well represent the aims

of our study. Therefore, we revised introduction in Ln 58-67.

Methods:

• At 6 weeks the mice are not adults so results from 6 and 12 weeks old mice should

be separated.

Because some previous studies of adult neurogenesis use young adult mice (6-8

weeks; e.g. Zhao et al., J Neurosci, 26: 3-11, 2006, Tashiro et al., Nature, 442: 929-

933, 2006), we followed the same in this study. However, we understand that some

researchers would think 6 weeks is not fully adult while more would agree if it is 2

months (although some may say 3 months). Therefore, we take the reviewer’s

suggestion. First we described the age of mice at the start of experiments more

clearly at Ln 80-83 in Materials and Methods. Second, we showed the results for

young adult (42-to-60 days old) and more mature adult mice (>60 days old)

separately in Figure 9. We have performed ANOVA including these age groups as a

between-subject factor and summarized statistical results in Table 6-8. We did this

only for Figure 5, 6, and 7A-F because we only used young adult mice in Figure 2, 3

and 7G-O and juvenile mice in Figure 4. For Figure 6 and 7A-F, we did not find any

significant effect of age. However, for Figure 5, three-way ANOVA detected

significant Age x Genotype x Housing interaction in the densities of BrdU+ and

BrdU+/Prox1+ cells. Although statistical power is low due to low sample number,

NR1 gene knockout seems to block enrichment induced increase in survival of new

neurons only in < 60-day-old but not > 60-day-old mice. Because the age-separated

analyses was not in our original experimental design, the sample size is low and the

result is not fully convincing. But the information would be useful for readers to use as

a guide for future experiments. We greatly appreciate the benefit of the reviewer’s 6

comment for an interesting result that came from the analyses that we would not

have done otherwise.

• Results obtained for male and female mice should be presented separately.

We have added Figure 8, which shows gender-separated graphs corresponding to all

graphs in Figure 2-4, 6 and 7. Please note that for Figure 5 we used females only as

described more in the answer to the next comment.

From the graphs in Figure 8, the effect of NR1 gene knockout seems to be generally

consistent between females and males. To support this observation, we performed

factorial ANOVA including gender as a between-subject factor (Table 1-5). In most

comparisons except six (see next paragraphs for more detail), no significant gender

difference was detected as supported by no significance in the main effect of gender

or interactions involving gender.

Four of such exceptions are results corresponding to 7 and 28 days for Figure 2F, 28

days for Figure 2H, and Figure 2J. For these exceptions, the main effects of Gender

and Genotype are significant while their interactions are not significant. Thus, the 7

effects of NR1 gene knockout occurred similarly in both females and males, although

the densities of BrdU+ and BrdU+/Prox1+ cells and survival rate are higher in

females irrespective of genotype. Another exception is a result corresponding to 7

days in Fig. 2H. Two-way ANOVA (Gender x Genotype) showed the main effect of

Genotype is significant (p = 0.028) although the t-test for the original analysis did not

detect this difference as significant (p = 0.070). This discrepancy may be because

there are variation between females and males (although not significant), which may

prevent between-genotype difference in the original analysis from reaching statistical

significance in the t-test when the varied data from two genders were analyzed

without being sorted. The last exception is the analysis corresponding to 2 weeks old

in Figure 4D; Gender x Genotype interaction is significant (Table 3). However, pre-planned simple effects tests with independent-sample t-test did not reach significance

for either female or male (Female: p = 0.153, Male: p = 0.128, independent t-tests,

two tailed). Thus, we did not detect significant difference between control and DGNR1KO mice in either gender, similarly to the original analysis in Figure 4D.

For survival analysis in Fig. 7B, we performed survival analysis stratified with gender.

We did not detect significant difference between control and DG-NR1KO mice.

In sum, we did not detect a clear gender difference in the effect of NR1 gene

knockout in any of the analyses in Figure 8 (although general gender differences

between genotypes were found in some measurements).8

• Were male and female mice exposed to the EE?

For the experiment with enriched environment, we used female mice only. We

performed the experiment by consulting a series of studies performed by Dr. Fred H.

Gage’s lab (Tashiro et al. J Neurosci, 27:3252-9, 2007; Zhao et al., J Comp Neurol,

522: 2756-66, 2015; Clemenson et al., Hippocampus, 25: 385-92, 2015), which used 9

only females for enriched environment. The reason why they used female mice only

is that their (and our) enriched environment include the housing with larger numbers

of mice. This requires to initially house adult mice in separate standard cages (e.g. 2

separate cages with 4 mice per cage). When initiating enriched housing, these mice

are now housed together. If they use male mice and house previously-separately housed mice together, often they start fighting each other, which makes it difficult to

interpret whether the observed effect is due to enriched environment or other

experience associated with fighting. This is why they (and we) always used female

mice for this type of enriched environment.

We now clearly describe the gender of mice in Ln 104.

• The critical values and ddl for the statistical analysis should be provided

We have added t and F values to all t-tests and ANOVAs, respectively, in the main

text. We did same for Table 1-8.

• Confocal analysis has not been used to determine the phenotype of BrdU cells.

This caveat should be discussed.

This is misunderstanding. We have used a confocal microscope for all colocalization

analysis, as described in Ln155-161 in the original manuscript. We apologize that our

description in Materials and Methods may have been confusing. To avoid this

misunderstanding, we revised description in Materials and Methods in Ln 186 and

187-193.

Results

• The model need to be better described if not published previously.

We understand that, by “The model", the reviewer meant the transgenic mouse line

(DG-NR1KO mice). This mouse line has been published (McHugh et al., Science,

317: 94-9, 2007). To make it clear that we used the same transgenic mouse line as

McHugh et al. (2007), we revised Ln 59-60 and Ln 72-73.

• I am not convinced by the lack of difference in Brdu-prox1 cells 7 days after the

injection (Fig 2F). They are at least 2 explanations: 1) inter-individual differences due

to sex difference and or low number of animals or to difference in the age of the

animals (6 to 12?), 2) results are biased by differences in neuronal differentiation.

Can the data of BrdU without prox1 be provided?

1)

Thank you for an excellent suggestion. To reflect gender difference in statistical

analyses, we performed two-way (Gender x Genotype) ANOVA (Figure 8, Table 1).

The main effects of Genotype are significant (p = 0.028) while interaction is not

significant (p = 0.829). This result indicates that, when gender difference is

considered, the difference between control and DG-NR1KO mice is significant

similarly for both gender. This result is now presented in Figure 8A and Table 1 (Note

that Figure 2F in the original manuscript is Figure 2H in the new manuscript).10

2)

According to the reviewer’s request, we now added the graphs for BrdU+ cell density

and proportion of BrdU+ cells expressing Prox1 as new Figure 2F and G. At 7 days,

BrdU+ density is significantly reduced (p = 0.0098, t(14) = 2.983, independent sample t-test, two tailed) while proportion of BrdU+ cells expressing Prox1 is not (p =

0.657, t(14) = -0.454, independent-sample t-test, two tailed).

• Why is there a difference in the volume of control mice in Fig3B and Fig 4F?

We apologize the lack of detailed description in the original manuscript. Fig 3B is total

volume in three analysed sections (both left and right hemispheres) and Fig 4F is

total volume in six analysed sections (both hemispheres). This is the major reason

why the number is different. We added this description in Materials and Methods (Ln

180-182) and in Figure legends in Ln 640 and 655. In addition, we made additional

revision in “Microscopy” section in Materials and Methods to include precise

information on how many sections were analysed in individual figures.

Discussion

• as the results obtained are different from those of Mc Hugh et al, a comparison of

the two genetic models is essential11

As we mentioned above, our DG-NR1KO mice are the same as the one in McHugh

et al. In addition, our result is not necessarily different from McHugh et al. As you can

see in Fig. 3, the reduction in the size of granule cell layer is ∼20% in DG-NR1KO

mice. Because the size of granule cell layer is highly variable among different

sections along the antero-posterior axis, the extent of reduction in DG-NR1KO mice

is smaller than variability among different sections. Therefore, the reduction in size

can be easily missed unless one systematically looks at many sections and quantifies

the volume as we did. The focus of McHugh et al. (2007) was not on the structure of

the dentate gyrus and no quantification was made.

• The authors concluded that the NR1 gene in granule cells is required for the normal

development of the granule. This is incorrect. Dentate granule neurons generated

during embryogenesis and the first 2 postnatal weeks do not seem to be impacted by

genetic NR1manipulation. But see Gould & Cameron 1997

The end of the first sentence in this reviewer’s comment seems to be lost and its

meaning is obscure. But we believe that the reviewer copied the sentence “the NR1

gene in granule cells is required for the normal development of the granule cell layer"

(Ln345-346 in the original manuscript). We agree that the development of the granule

cell layer during embryogenesis and the first 2 postnatal weeks seems to be normal.

But we found that the size of granule cell layer is small and neurogenesis is reduced

after the postnatal age of 3 weeks in DG-NR1KO mice. These results suggest that

"the NR1 gene in granule cells is required for the normal development of the granule

cell layer”. This is a reasonable interpretation.

By the way, although this is slightly off the topic, as we mentioned in Ln 54 (in the

original manuscript), McHugh showed NR1 gene knockout starts between 1.5 and 4

weeks; they detect reduction of NR1 transcripts at 4 weeks, but not at 1.5 weeks.

This is likely to be the reason why we did not detect any difference at postnatal 2

weeks. Then, our result of no effect at the postnatal age of 2 weeks is not evidence

that the development of the granule cell layer before 2 weeks or granule cells born

before 2 weeks are not dependent on the NMDA receptors. Rather a different

manipulation method is required to examine this issue.

• Line 349: here (and not in the intro) discuss McHugh et al. !

We have added sentences to discuss McHugh et al. (2007) in Ln 497-501 (after Ln

349 in the original manuscript).

• 353: the term postnatal neurogenesis is confusing. Adult neurogenesis also occurs

postnatally

We do not fully agree with this comment. When one say ‘postnatal’, it usually means

a relatively short period after birth but not for a long period after birth. It always does

not include adulthood. For example, “postnatal care” almost always refers to care

during relatively a short of period after the birth, and never includes care after the

baby becomes adult.12

Nonetheless, to avoid confusion, we revised the texts that may be confusing at

multiple places. (e.g., ’postnatal dentate gyrus’ -> 'postnatal, developing dentate

gyrus' and 'the postnatal period' -> 'the first weeks of postnatal period').

• Paragraph line 385. The data of Gould&Cameron 1997 deserve to be included in

the discussion.

Gould & Cameron 1997 showed that an NMDA receptor antagonist, CGP43487,

increases cell proliferation in the dentate gyrus. We cited Gould & Cameron 1997

together with Cameron et al., 1995 in Ln 539. Because the paragraph is specifically

about a role of NMDA receptor in neurogenesis/cell proliferation but not behaviour,

behavioural results of Gould & Cameron 1997 are not mentioned here.

• The behavioural test is not clearly justified.

We revised the text to “Previous studies suggested that adult neurogenesis has a

role in anxiety-related behaviors (Jacobs et al., 2000; Santarelli et al., 2003; Surget et

al., 2011). Therefore, we examined the behaviour of adult, control and DG-NR1KO

mice (n = 22 and 21 mice, respectively) in the novelty-suppressed feeding test, open

field test and elevated plus maze (Santarelli et al., 2003; Walf and Frye, 2007;

Seibenhener and Wooten, 2015).” in Ln 370-374.

• The results seem to indicate a deficit in anxiety like-responses. Another would be

welcome to confirm this data (Plus maze)

Following the reviewer's suggestion, we added open field test (Figure 7G-J) and

elevated plus maze (Figure 7K-O). We did not find significant difference in travelled

distance and time spent in peripheral/inner zones in open field test and %Time in and

%Entries to open arms in elevated plus maze. However, DG-NR1KO mice showed

significant decrease in fecal counts during open field test, which has been used for

an indication of anxiety level (Seibenhener et al., J Vis Exp, 96: e52434, 2015).

Lower fecal counts has been considered to reflect lower anxiety level. Thus, from the

three anxiety-related tasks, two measures (%time in peripheral/centre zone in

novelty-suppressed feeding test and fecal counts in open field test) suggest lower

anxiety level in DG-NR1KO mice while other measures, which has been considered

as main indices for anxiety level in these tasks, did not show difference. Therefore,

while we continue to be reluctant to conclude the effect on anxiety level, the

additional results have made more convincing our original position that there is some

behavioural abnormality in DG-NR1KO mice.

Figure 7G-O1

## References

[B51] Åmellem I, Suresh S, Chang CC, Tok SSL, Tashiro A (2017) A critical period for antidepressant-induced acceleration of neuronal maturation in adult dentate gyrus. Transl Psychiatry 7:e1235. 28925998 10.1038/tp.2017.208 PMC5639251

[B1] Bannerman DM, Bus T, Taylor A, Sanderson DJ, Schwarz I, Jensen V, Hvalby Ø, Rawlins JN, Seeburg PH, Sprengel R (2012) Dissecting spatial knowledge from spatial choice by hippocampal NMDA receptor deletion. Nat Neurosci 15:1153–1159. 10.1038/nn.3166 22797694 PMC3442238

[B2] Bear MF, Cooper LN, Ebner FF (1987) A physiological basis for a theory of synapse modification. Science 237:42–48. 10.1126/science.3037696 3037696

[B3] Bruel-Jungerman E, Davis S, Rampon C, Laroche S (2006) Long-term potentiation enhances neurogenesis in the adult dentate gyrus. J Neurosci 26:5888–5893. 10.1523/JNEUROSCI.0782-06.2006 16738230 PMC6675234

[B4] Cameron HA, Woolley CS, McEwen BS, Gould E (1993) Differentiation of newly born neurons and glia in the dentate gyrus of the adult rat. Neuroscience 56:337–344. 10.1016/0306-4522(93)90335-d 8247264

[B5] Cameron HA, McEwen BS, Gould E (1995) Regulation of adult neurogenesis by excitatory input and NMDA receptor activation in the dentate gyrus. J Neurosci 15:4687–4692. 7790933 10.1523/JNEUROSCI.15-06-04687.1995 PMC6577705

[B6] Constantine-Paton M, Cline HT, Debski E (1990) Patterned activity, synaptic convergence, and the NMDA receptor in developing visual pathways. Annu Rev Neurosci 13:129–154. 10.1146/annurev.ne.13.030190.001021 2183671

[B7] Czerniawski J, Ree F, Chia C, Ramamoorthi K, Kumata Y, Otto TA (2011) The importance of having Arc: expression of the immediate-early gene Arc is required for hippocampus-dependent fear conditioning and blocked by NMDA receptor antagonism. J Neurosci 31:11200–11207. 10.1523/JNEUROSCI.2211-11.2011 21813681 PMC6623359

[B8] Encinas JM, Sierra A, Valcárcel-Martín R, Martín-Suárez S (2013) A developmental perspective on adult hippocampal neurogenesis. Int J Dev Neurosci 31:640–645. 10.1016/j.ijdevneu.2013.04.001 23588197

[B9] Ewald RC, Cline HT (2009) NMDA receptors and brain development. In: Biology of the NMDA receptor (Van Dongen AM, ed). Boca Raton: CRC Press/Taylor & Francis. 21204419

[B10] Ge S, Yang CH, Hsu KS, Ming GL, Song H (2007) A critical period for enhanced synaptic plasticity in newly generated neurons of the adult brain. Neuron 54:559–566. 10.1016/j.neuron.2007.05.002 17521569 PMC2040308

[B11] Gould E, Cameron HA (1997) Early NMDA receptor blockade impairs defensive behavior and increases cell proliferation in the dentate gyrus of developing rats. Behav Neurosci 111:49–56. 10.1037//0735-7044.111.1.49 9109623

[B12] Gould E, Cameron HA, McEwen BS (1994) Blockade of NMDA receptors increases cell death and birth in the developing rat dentate gyrus. J Comp Neurol 340:551–565. 10.1002/cne.903400408 7911808

[B13] Gould E, McEwen BS, Tanapat P, Galea LA, Fuchs E (1997) Neurogenesis in the dentate gyrus of the adult tree shrew is regulated by psychosocial stress and NMDA receptor activation. J Neurosci 17:2492–2498. 9065509 10.1523/JNEUROSCI.17-07-02492.1997 PMC6573503

[B14] Guzowski JF, McNaughton BL, Barnes CA, Worley PF (1999) Environment-specific expression of the immediate-early gene Arc in hippocampal neuronal ensembles. Nat Neurosci 2:1120–1124. 10.1038/16046 10570490

[B15] Guzowski JF, Lyford GL, Stevenson GD, Houston FP, McGaugh JL, Worley PF, Barnes CA (2000) Inhibition of activity-dependent arc protein expression in the rat hippocampus impairs the maintenance of long-term potentiation and the consolidation of long-term memory. J Neurosci 20:3993–4001. 10818134 10.1523/JNEUROSCI.20-11-03993.2000 PMC6772617

[B16] Guzowski JF, Setlow B, Wagner EK, McGaugh JL (2001) Experience-dependent gene expression in the rat hippocampus after spatial learning: a comparison of the immediate-early genes Arc, c-fos, and zif268. J Neurosci 21:5089–5098. 10.1523/JNEUROSCI.21-14-05089.2001 11438584 PMC6762831

[B17] Haberny KA, Paule MG, Scallet AC, Sistare FD, Lester DS, Hanig JP, Slikker W Jr (2002) Ontogeny of the N-methyl-D-aspartate (NMDA) receptor system and susceptibility to neurotoxicity. Toxicol Sci 68:9–17. 10.1093/toxsci/68.1.9 12075105

[B18] Hansen HH, Briem T, Dzietko M, Sifringer M, Voss A, Rzeski W, Zdzisinska B, Thor F, Heumann R, Stepulak A, Bittigau P, Ikonomidou C (2004) Mechanisms leading to disseminated apoptosis following NMDA receptor blockade in the developing rat brain. Neurobiol Dis 16:440–453. 10.1016/j.nbd.2004.03.013 15193300

[B19] Ikonomidou C, Bosch F, Miksa M, Bittigau P, Vöckler J, Dikranian K, Tenkova TI, Stefovska V, Turski L, Olney JW (1999) Blockade of NMDA receptors and apoptotic neurodegeneration in the developing brain. Science 283:70–74. 10.1126/science.283.5398.70 9872743

[B20] Jacobs BL, Van Praag H, Gage FH (2000) Adult brain neurogenesis and psychiatry: a novel theory of depression. Mol Psychiatry 5:262–269. 10.1038/sj.mp.4000712 10889528

[B21] Kempermann G, Kuhn HG, Gage FH (1997) More hippocampal neurons in adult mice living in an enriched environment. Nature 386:493–495. 10.1038/386493a0 9087407

[B22] Li G, Pleasure SJ (2005) Morphogenesis of the dentate gyrus: what we are learning from mouse mutants. Dev Neurosci 27:93–99. 10.1159/000085980 16046842

[B23] McHugh TJ, Jones MW, Quinn JJ, Balthasar N, Coppari R, Elmquist JK, Lowell BB, Fanselow MS, Wilson MA, Tonegawa S (2007) Dentate gyrus NMDA receptors mediate rapid pattern separation in the hippocampal network. Science 317:94–99. 10.1126/science.1140263 17556551

[B24] Mu Y, Zhao C, Toni N, Yao J, Gage FH (2015) Distinct roles of NMDA receptors at different stages of granule cell development in the adult brain. Elife 4:e07871. 10.7554/eLife.07871 26473971 PMC4608052

[B25] Oreland S, Nylander I, Pickering C (2010) Prolonged maternal separation decreases granule cell number in the dentate gyrus of 3-week-old male rats. Int J Dev Neurosci 28:139–144. 10.1016/j.ijdevneu.2009.12.005 20079421

[B26] Overstreet LS, Hentges ST, Bumaschny VF, De Souza FSJ, Smart JL, Santangelo AM, Low MJ, Westbrook GL, Rubinstein M (2004) A transgenic marker for newly born granule cells in dentate gyrus. J Neurosci 24:3251–3259. 10.1523/JNEUROSCI.5173-03.2004 15056704 PMC6730035

[B27] Pickering C, Gustafsson L, Cebere A, Nylander I, Liljequist S (2006) Repeated maternal separation of male Wistar rats alters glutamate receptor expression in the hippocampus but not the prefrontal cortex. Brain Res 1099:101–108. 10.1016/j.brainres.2006.04.136 16784730

[B52] Plath N, Ohana O, Dammermann B, Errington ML, Schmitz D, Gross C, Mao X, Engelsberg A, Mahlke C, Welzl H, Kobalz U, Stawrakakis A, Fernandez E, Waltereit R, Bick-Sander A, Therstappen E, Cooke SF, Blanquet V, Wurst W, Salmen B et al. (2006) Arc/Arg3.1 is essential for the consolidation of synaptic plasticity and memories. Neuron 52:437–444. 17088210 10.1016/j.neuron.2006.08.024

[B28] Revest JM, Dupret D, Koehl M, Funk-Reiter C, Grosjean N, Piazza PV, Abrous DN (2009) Adult hippocampal neurogenesis is involved in anxiety-related behaviors. Mol Psychiatry 14:959–967. 10.1038/mp.2009.15 19255582

[B29] Roceri M, Hendriks W, Racagni G, Ellenbroek BA, Riva MA (2002) Early maternal deprivation reduces the expression of BDNF and NMDA receptor subunits in rat hippocampus. Mol Psychiatry 7:609–616. 10.1038/sj.mp.4001036 12140784

[B30] Santarelli L, Saxe M, Gross C, Surget A, Battaglia F, Dulawa S, Weisstaub N, Lee J, Duman R, Arancio O, Belzung C, Hen R (2003) Requirement of hippocampal neurogenesis for the behavioral effects of antidepressants. Science 301:805–809. 10.1126/science.1083328 12907793

[B31] Seibenhener ML, Wooten MC (2015) Use of the open field maze to measure locomotor and anxiety-like behavior in mice. J Vis Exp. Advance online publication. Retrieved 6 Feb, 2015. doi: 10.3791/52434. PMC4354627 25742564

[B32] Snyder JS, Soumier A, Brewer M, Pickel J, Cameron HA (2011) Adult hippocampal neurogenesis buffers stress responses and depressive behaviour. Nature 476:458–461. 10.1038/nature10287 21814201 PMC3162077

[B33] Steward O, Wallace CS, Lyford GL, Worley PF (1998) Synaptic activation causes the mRNA for the IEG Arc to localize selectively near activated postsynaptic sites on dendrites. Neuron 21:741–751. 10.1016/S0896-6273(00)80591-7 9808461

[B34] Surget A, Tanti A, Leonardo ED, Laugeray A, Rainer Q, Touma C, Palme R, Griebel G, Ibarguen-Vargas Y, Hen R, Belzung C (2011) Antidepressants recruit new neurons to improve stress response regulation. Mol Psychiatry 16:1177–1188. 10.1038/mp.2011.48 21537331 PMC3223314

[B35] Tashiro A, Zhao C, Gage FH (2006a) Retrovirus-mediated single-cell gene knockout technique in adult newborn neurons in vivo. Nat Protoc 1:3049–3055. 10.1038/nprot.2006.473 17406567

[B36] Tashiro A, Sandler VM, Toni N, Zhao C, Gage FH (2006b) NMDA-receptor-mediated, cell-specific integration of new neurons in adult dentate gyrus. Nature 442:929–933. 10.1038/nature05028 16906136

[B37] Tashiro A, Makino H, Gage FH (2007) Experience-specific functional modification of the dentate gyrus through adult neurogenesis: a critical period during an immature stage. J Neurosci 27:3252–3259. 10.1523/JNEUROSCI.4941-06.2007 17376985 PMC6672473

[B38] Tashiro A, Zhao C, Suh H, Gage FH (2015) Imaging newborn granule cells in fixed sections. Cold Spring Harb Protoc 2015:932–933. 10.1101/pdb.prot086389 26430256

[B39] Walf AA, Frye CA (2007) The use of the elevated plus maze as an assay of anxiety-related behavior in rodents. Nat Protoc 2:322–328. 10.1038/nprot.2007.44 17406592 PMC3623971

